# Structure of SgK223 pseudokinase reveals novel mechanisms of homotypic and heterotypic association

**DOI:** 10.1038/s41467-017-01279-9

**Published:** 2017-10-27

**Authors:** Onisha Patel, Michael D. W. Griffin, Santosh Panjikar, Weiwen Dai, Xiuquan Ma, Howard Chan, Celine Zheng, Ashleigh Kropp, James M. Murphy, Roger J. Daly, Isabelle S. Lucet

**Affiliations:** 1 The Walter and Eliza Hall Institute of Medical Research, Parkville, VIC 3052 Australia; 2 Department of Medical Biology, University of Melbourne, Parkville, VIC 3052 Australia; 3 Department of Biochemistry and Molecular Biology, Bio21 Molecular Science and Biotechnology Institute, University of Melbourne, Parkville, VIC 3052 Australia; 4 Australian Synchrotron, Clayton, VIC 3168 Australia; 5 Department of Biochemistry and Molecular Biology, Level 1, Building 77, Monash University, Clayton, VIC 3800 Australia; 6 Cancer Program, Biomedicine Discovery Institute, Monash University, Clayton, VIC 3800 Australia

## Abstract

The mammalian pseudokinase SgK223, and its structurally related homologue SgK269, are oncogenic scaffolds that nucleate the assembly of specific signalling complexes and regulate tyrosine kinase signalling. Both SgK223 and SgK269 form homo- and hetero-oligomers, a mechanism that underpins a diversity of signalling outputs. However, mechanistic insights into SgK223 and SgK269 homo- and heterotypic association are lacking. Here we present the crystal structure of SgK223 pseudokinase domain and its adjacent N- and C-terminal helices. The structure reveals how the N- and C-regulatory helices engage in a novel fold to mediate the assembly of a high-affinity dimer. In addition, we identified regulatory interfaces on the pseudokinase domain required for the self-assembly of large open-ended oligomers. This study highlights the diversity in how the kinase fold mediates non-catalytic functions and provides mechanistic insights into how the assembly of these two oncogenic scaffolds is achieved in order to regulate signalling output.

## Abstract (web summary)

Pseudokinases lack kinase activity, yet they impact cellular physiology through the regulation of bona fide signaling kinases. Here the authors describe the structure of the SgK223 pseudokinase and its adjacent domains, and identify regulatory interfaces required for self-assembly and downstream signaling.

## Introduction

Pseudokinases have recently emerged as crucial regulators of many cellular functions. Despite a lack of catalytic activity, the pseudokinase (PsK) domain structure resembles that of classical kinases, enabling pseudokinases to undergo protein–protein interactions to disseminate or coordinate signal outputs. Their functions vary from allosteric regulators that fine-tune kinase catalytic activities^1–6^ to molecular switches that propagate signal output^7^ and scaffolds that nucleate the assembly of signalling complexes^8–11^.

Sugen kinase 223 (SgK223) (also known as Pragmin) and SgK269 (also known as PEAK1) are closely related proteins of 149 and 193 kDa, respectively. Both are classified as pseudokinases due to the substitution of critical residues within highly conserved motifs known to be essential for kinase activity^12, 13^, and both are predicted to function as scaffolds^10, 14–16^. They share a domain organisation that consists of an N-terminal region predicted to be partly folded, a largely unstructured central PEST region and a C-terminal PsK domain flanked either side by predicted helical regulatory domains^10^. The PEST central region harbours tyrosine phosphorylation sites that specifically recruit SH2 and PTB domain-containing effectors, however the function of the conserved N- and C-terminal regions, including the PsK domain, remains largely unknown.

**Fig. 1:**
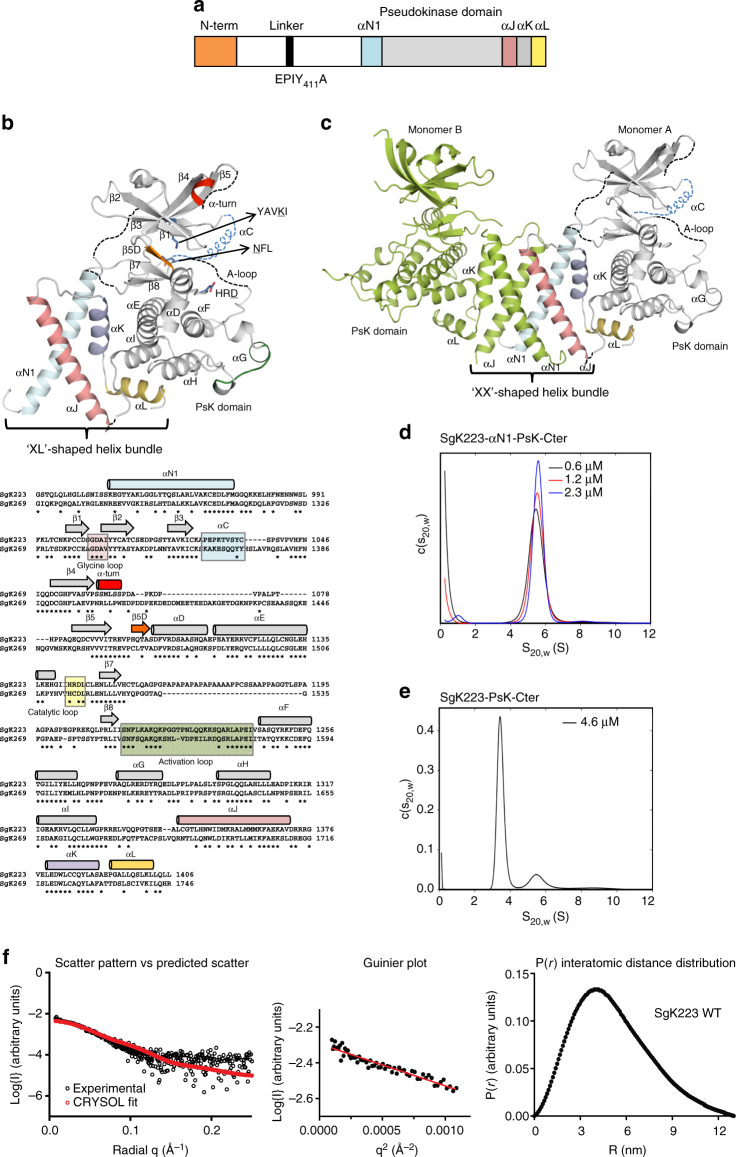
Structure of SgK223-αN1-PsK-Cter. **a** Schematic representation of SgK223. Y411 is phosphorylated by Csk, and also serves as docking site for this kinase. **b** Structure of SgK223-αN1-PsK-Cter monomer (top) and sequence alignment of SgK223 and SgK269 and secondary structure elements as determined from the SgK223-αN1-PsK-Cter crystal structure (bottom). αN1, cyan; PsK domain, grey; αJ, salmon; αK violet; αL yellow; catalytic residues are shown in sticks and underlined; αC is modelled in blue dashed lines. **c** Structure of SgK223-αN1-PK-Cter dimer. Monomer A colour coding as in **b**; monomer B shown in green. **d** Continuous standardized sedimentation coefficient [*c*(*s*_20,w_)] distributions for SgK223-αN1-PsK-Cter at concentrations of 0.6 μM (black line), 1.2 μM (red line) and 2.3 μM (blue line). **e** *c*(*s*_20,w_) distribution for SgK223-PsK-Cter at a concentration of 4.6 μM. **f** Left: overlay of experimental scattering data (black circles) and scattering profile calculated using CRYSOL (red). Middle: Guinier plot indicating that aggregates do not measurably contribute to the scattering profile. Right: Interatomic distance distributions. See also Supplementary Fig. 1

SgK223 was first identified as an effector of Rnd2, a member of the Rho family of GTPases, and shown to regulate neuron outgrowth by functioning as a RhoA activator^17^. More recently, SgK223 has emerged as a protein scaffold that promotes Src family kinase (SFK) signalling by regulating the subcellular localisation of C-terminal Src kinase (Csk), a negative regulator of these kinases^14^. Once phosphorylated on tyrosine residue Y411, SgK223 binds and sequesters Csk^14^, which prevents Csk-mediated SFK phosphorylation and inactivation at the plasma membrane, leading to sustained SFK activation. A recent study found SgK223 and Csk were co-localized at focal adhesions where they regulate cell morphology and motility^18^. Consistent with these findings, SgK223 has emerged as an important regulator of cell morphology, migration and invasion. Overexpression of SgK223 leads to a more elongated, fibroblastic morphology^18, 19^ and enhances migratory and invasive potential^19^. In addition, SgK223 is implicated in progression of specific cancers, being required for Src-induced invasion of colon cancer cells^20^, and upregulated during pancreatic cancer development, where it acts a positive regulator of Stat3^19^.

The related protein, SgK269/PEAK1, is also implicated in regulation of cell migration. SgK269 localises to the pseudopodia of migrating cells, associates with the actin cytoskeleton and focal adhesions and promotes cell motility^15^. It plays a key role in regulating the maturation and disassembly of focal adhesions, a function that is dependent on SgK269 phosphorylation at Y665 and SFK activity^21^. During EGF signalling via the scaffold Shc1, SgK269 mediates a critical switch in signal output, binding Shc1 via SgK269 Y1188 and promoting migratory/invasive responses^16^. However, SgK269 can also promote mitogenesis by recruiting Grb2 to Y635 and activating the Ras pathway^22^. Reflecting these different functional properties, overexpression of SgK269 in mammary epithelial cells promotes a partial epithelial–mesenchymal transition, and aberrant growth and morphogenesis^22^. As with SgK223, SgK269 is implicated in human malignancy and is overexpressed in a subset of breast, colon and pancreatic cancers^15, 22, 23^.

In a recent study, we demonstrated that SgK269 and SgK223 are both capable of homo-dimerization, an event in part driven by a 43 amino-acid helix (termed αN1) directly upstream of the PsK domain^10^. We also reported hetero-oligomerization between SgK223 and SgK269, a mechanism that is dependent on the presence of the αN1 helix and the C-terminal containing PsK region and demonstrated the importance of SgK223–SgK269 hetero-oligomerization in promoting cell migration and STAT3 activation^10^. However, the molecular basis for these interactions has remained unclear. To gain further insight into the molecular mechanism that drives SgK223 dimerization and oligomerization functions, we solved the crystal structure of the PsK domain of SgK223 and its adjacent regulatory domains. Our structure reveals that the αN1 helix and the C-terminal domain fold together in a unique helix bundle that drives SgK223 homodimerization. In addition, our structure uncovers an unexpected feature of the SgK223 PsK domain where the αG helix forms a critical interaction interface that promotes oligomerization. Together, our structural data, combined with mutational analysis, biochemical and biophysical and cellular studies, provide a detailed understanding of the mechanism that drive homo- and heterotypic association of these two pseudokinase scaffolds, and suggests the importance of higher-order scaffold assembly in regulating signal output.

## Results

### SgK223 dimerizes via the PsK domain-flanking regions

To fully understand the structural determinants that drive SgK223 dimerization, we solved the crystal structure of a construct (SgK223- αN1-PsK-Cter) that encompasses the PsK domain and the adjacent N- and C-regulatory domains (Fig. 1a) using the multi-wavelength anomalous dispersion method. The structure was refined against native data to 2.95 Å resolution. SgK223 crystallized in space group P4_3_2_1_2 with one molecule in the asymmetric unit (Table 1). Within a monomer, the PsK domain of SgK223 adopts a typical kinase-like fold and the N-terminal helix αN1 directly interacts with the C-terminal helices αJ and αK, while αL helix slides under αI helix of the PsK domain (Fig. 1b) revealing an unusual ‘XL’-shaped helix bundle. While the asymmetric unit contains a monomer, the P4_3_2_1_2 crystal form revealed a two-fold symmetric dimer with an extensive buried surface area (BSA) of 1620 Å^2^. SgK223 dimer formation is mediated by the regulatory helices from the two monomers with the αN1 and the αJ helices from one monomer interacting with the corresponding αJ and αN1 helices from the second monomer to form a unique ‘XX’-shaped four-helix bundle at the ‘core’ of the dimer and positioning the PsK domains peripherally to form the ‘shell’ of the dimer (Fig. 1c). This distinctive αN1/αJ helix bundle reveals an extensive interaction footprint consistent with a large BSA and highlights the rigidity of the dimeric packing (Fig. 1c and Supplementary Fig. 1). Within the ‘XX’ fold, αN1 and αJ arrange in a left-handed parallel fashion reminiscent of the arrangement of helices of leucine zipper GCN4, packing against each other in a ‘knobs-into-holes’ arrangement, further accentuating the shape complementarity of the dimeric interface (Supplementary Fig. 1). This packing is consistent with our previous data showing that the removal of αN1 leads to a monomeric form of SgK223^10^.

**Table 1:** Data collection, phasing and refinement statistics for MAD (SeMet) structures

	SgK223		SgK223		
	Native		Se-met		
*Data collection*					
Space group	P4_3_2_1_2		P4_3_2_1_2		
*Cell dimensions*					
*a*, *b*, *c* (Å)	144.68, 144.68, 47.44		144.75, 144.75, 47.37		
*α*, *β*, *γ* (°)	90, 90, 90		90, 90, 90		
		Remote	Remote	Inflection	Inflection
Wavelength	0.9537	0.9537	0.9537	0.9796	0.9796
Resolution (Å)	20–2.95 (3.13-2.95)*	20–3.53 (3.75-3.53)*	20–3.41 (3.61-3.41)*	20–3.38 (3.58-3.38)*	20–3.38 (3.58-3.38)*
*R*_merge_	10.2 (65.9)	23.9 (75.8)	16.2 (68.5)	18.3 (77.5)	14.4 (61.7)
*I*/σ*I*	18.62 (3.85)	8.25 (2.96)	13.68 (3.47)	12.96 (3.21)	13.21 (3.42)
Completeness (%)	99.7 (98.0)	99.8 (98.9)	99.7 (98.8)	99.7 (98.6)	99.9 (99.7)
Redundancy	8.50 (8.38)	7.69 (7.69)	7.77 (7.70)	7.75 (7.65)	7.74 (7.67)
*Refinement*					
Resolution (Å)	20–2.95 (3.13-2.95)*	20–3.53 (3.75-3.53)*	20–3.41 (3.61-3.41)*	20–3.38 (3.58-3.38)*	20–3.38 (3.58-3.38)*
No. of reflections	171,294 (26,847)	90,672 (14,470)	102,283 (16,160)	104,515 (16,372)	104,831 (16,468)
*R*_work_/*R*_free_	0.222/0.262				
No. of atoms					
Protein	2718				
Ligand/ion	—				
Water	9				
B-factors					
Protein	59.4				
Ligand/ion	—				
Water	57.9				
R.m.s. deviations					
Bond lengths (Å)	0.003				
Bond angles (°)	0.652				

Each structure was determined from one crystal

*Values in parentheses are for highest resolution

To further confirm the dimerization state of SgK223, we assessed the behaviour of SgK223 using analytical ultracentrifugation. Sedimentation velocity experiments with SgK223-αN1-PsK-Cter at concentrations between 0.6 and 74.0 μM indicated that at concentrations below 2.3 μM the protein sedimented as a reasonably homogeneous species with a sedimentation coefficient of ~5.6 s (Fig. 1d and Supplementary Fig. 1), consistent with a dimer. Weight average sedimentation coefficients calculated for 0.6, 1.2 and 2.3 μM of SgK223-αN1-PsK-Cter were 5.55, 5.62 and 5.66 s, corresponding to molecular weights (MW) of 120, 113 and 96.5 kDa, respectively, provided further evidence for a dimeric structure in solution (monomer MW, 54 kDa). The relative stability of the weight average sedimentation coefficient over this concentration range suggests that the dimer > monomer dissociation constant is in the mid- to low-nanomolar range. In contrast, at concentrations above 4.6 μM, the *c*(*s*_20,w_) distributions shifted towards higher sedimentation coefficients, with a weight average sedimentation coefficient of 7.83 s at 74.0 μM, consistent with formation of tetramer and higher-order oligomers (Supplementary Fig. 1). Sedimentation velocity experiments with SgK223-PsK-Cter construct without αN1, provided *c*(*s*_20,w_) distributions with a major peak around 3.4 s (Fig. 1e) consistent with a monomer and our previous data showing that this construct eluted as a monomer by analytical size-elution chromatography (SEC)^10^.

To verify that the homodimeric conformation observed in the crystal is representative of the in solution structure of SgK223, we performed Small-Angle X-ray Scattering (SAXS). The maximum particle dimension (*D*_max_) was measured as 130 Å, consistent with the dimensions of the SgK223 dimer crystal structure. Furthermore, the experimental scatter pattern was consistent with the theoretical scatter calculated based on the crystal structure coordinates (Fig. 1f; *χ* = 0.43), thereby verifying the crystallized dimer is akin to the in solution conformation.

Taken together, the AUC and SAXS data confirm that SgK223 exists as a dimer in solution, validating the crystallized dimer conformation and confirming the structural role of regulatory helices in stabilizing the dimer interface.

### SgK223 pseudokinase domain has unique features

**Fig. 2:**
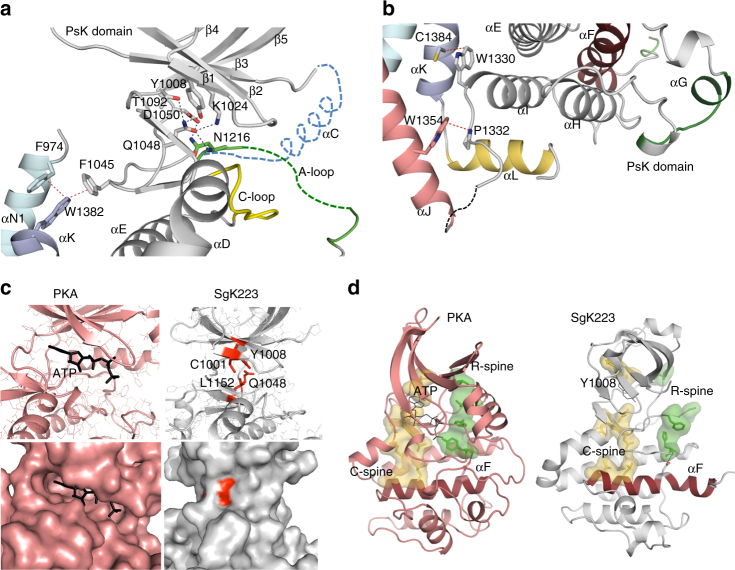
The unique features of SgK223 PsK domain. **a** Interaction between N-lobe of the PsK domain and the dimerization domain. **b** Interaction between the C-lobe of the PsK domain and the dimerization domain. αN1, cyan; PsK domain, grey; αJ, salmon; αK violet; αL yellow; A-loop, green; C-loop, yellow; αC is modelled in blue dashed lines. **c** Comparison of the nucleotide binding site of PKA (left) and SgK223 (right). PKA, salmon; ATP, black; SgK223, grey, residues occupying the ATP-binding sites shown in red. **d** Location of C- and R-spines on PKA and SgK223. C-spine, yellow; R-spine, green; αF, dark red

The PsK domain of each monomer adopts a canonical bilobal kinase fold, which consists of an N-lobe composed of five stranded β-sheets (β1–β5) that connect to a larger α-helical C-lobe through a hinge region. Despite a common overall kinase architecture fold, striking structural differences are seen within the N-lobe. It contains a α-turn (SSMLS) between the β4 and β5 strands, a feature also seen in elF2α kinases^24^, despite a lack of sequence similarity (Fig. 1b and Supplementary Fig. 2). This α-turn marks the start of an insertion loop unique to SgK223 and SgK269, however, its electron density is not resolved and its structural role remains unclear. In addition, the N-lobe has an extra β5D strand that lies parallel to the β7 and β8 strands and connects the β5 strand to the αD helix (Fig. 1b). The most notable feature is the absence of a structured αC helix, a critical feature in bona fide kinases. The predicted αC helix sequence in SgK223 is shorter and poorly conserved (Fig. 1b and Supplementary Fig. 2). Particularly, the αC helix is missing the conserved glutamate, which forms the canonical β3−Lys/αC-Glu salt-bridge interaction, a structural feature of a catalytically active kinase conformation. Instead, in SgK223, the conserved β3 Lys1024 from the YAVK motif makes H-bond interactions with Gln1048 from the loop preceding the β4 strand. Gln1048 in turn makes H-bond interaction with Tyr1008 from the β2 strand (Fig. 2a) and the predicted gate-keeper residue Thr1092. This unusual interaction locks the orientation of the N-lobe of the PsK domain with respect to the C-lobe, clearly influencing the relative positions of the key residues known to be crucial for catalytic activity in conventional protein kinases. The N-lobe is further stabilized in this position by π-stacking interactions mediated by the triad of Phe1045 from the β3–β4 loop with Trp1382 from the αK and Phe974 of αN1 helix (Fig. 2a). The β3-β4 loop provides therefore a critical anchor point for the PsK domain to the N- and C-terminal regulatory domains. The start of the activation loop (A-loop) is characterized by SNF instead of the canonical DFG motif known to be crucial for the coordination of cations in bona fide kinases^25^ (Fig. 2b). The SNF and the APE motifs are the only portion of the A-loop that are resolved in the structure. The catalytic loop (HRD_1445_LCLEN) adopts a conformation similar to bona fide kinases (Figs. 1b and 2a). The conserved tryptophan, Trp1330, at the end of the PsK domain, packs against the backbone of the αK helix and the loop that connects the end of the PsK domain and the start of the C-terminal helices. This interaction is stabilized by the bulky side chain of Trp1354 from the αJ helix, keeping the C-lobe of the PsK domain tightly anchored to the dimerization domain (Fig. 2b).

### SgK223 has a degraded ATP-binding pocket

We previously determined by thermal shift assay (TSA) that SgK223 did not detectably bind nucleotides^12^. The structure of SgK223 clearly demonstrates an absence of an ATP-binding cavity, which is instead occupied by the side chains of residues Tyr1008, Cys1001, Gln1048 and Leu1152 (Fig. 2c). The hydrophobic interactions between Phe1045 (β3-β4 loop, N-lobe PsK), Trp1382 (αK) and Phe 974 (αN1), the interaction between Trp1354 (αJ) and Pro1332 (C-lobe PsK) and the triad interaction between Lys1024 (β3), Gln1048 (β3-β4 loop) and Tyr1008 (β2) all highly contribute to the rigidity of the positioning of the N-lobe relative to the C-lobe and the loss of the ATP binding site (Fig. 2a). As a result of these modifications, the critical residues that make up the regulatory (R-) and catalytic (C-) spines are no longer aligned in SgK223 when compared to the canonical, active structure of the archetypal kinase PKA, suggesting a severely impaired catalytic core (Fig. 2d). Interestingly, the residues that occupy the ATP binding cavity in SgK223 are conserved in SgK269 (Fig. 1b) predicting that SgK269 is also devoid of any nucleotide binding ability, consistent with our earlier findings using TSA^12^.

### The adjacent helices dictate SgK223 PsK domain conformation

**Fig. 3:**
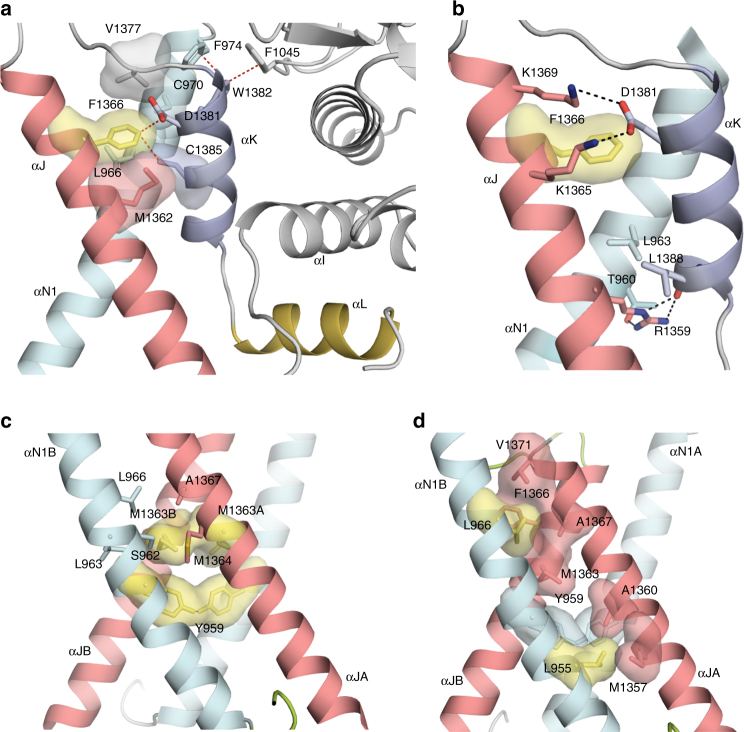
SgK223 forms a high-affinity dimer. **a**, **b** Interaction between αN1, αJ and αK helices and the PsK domain. **c** Met1363 and Tyr959 side chains are buried within the hydrophobic core at the ‘XX’ dimer interface between αN1 and αJ helices. **d** Hydrophobic interaction surrounding Leu955 and Leu966 at the dimer interface. αN1, cyan; PK domain, grey; αJ, salmon; αK violet; αL yellow; h-bond and van der Waals interactions are shown in black and red dashed lines, respectively

Within a monomer of SgK223, the regulatory helices αN1, αJ and αK are stabilized by a number of polar and non-polar interactions. Importantly, αJ Phe1366 makes direct interactions with αK Asp1381 and Cys1385 and is buried in a hydrophobic pocket formed by neighbouring Met1362 and Val1377 located in the αJ-αK connecting loop, and αN1 Cys970 and Leu966. In addition, αK Asp1381 further stabilizes the αJ-αK connection by making a charged interaction with αJ surface exposed Lys1365 and Lys1369 (Fig. 3a, b). Interestingly, the side chain of Arg1359 from the αJ helix protrudes from the junction of αN1, αJ and αK such that it makes extensive interactions with αN1 residues Thr960 and Leu963 as well as αK Leu1388. The guanidinium group of Arg1359 makes H-bond interactions with the hydroxyl group of αN1 Thr960 as well as the main chain carbonyl oxygen of αK Leu1388 (Fig. 3b), thus stabilizing the αN1-αJ-αK junction. Lastly, while the αL helix interacts with αI of the PsK domain, this interaction is dominated by backbone, rather than side-chain, interactions. Together, these interactions, combined with the hydrophobic interactions of the Phe1045 (N-lobe), Trp1382 (αK) and Phe974 (αN1) triad (Fig. 2a), contribute to the structural integrity of the N- and C-regulatory helices and their anchoring to the PsK domain.

### Hydrophobic hot spots drive the SgK223 high-affinity dimer

The dimer interface of SgK223 observed in the crystal structure, formed by the unique arrangement of αN1 and αJ into a ‘XX’-shaped helices bundle, is dominated by hydrophobic interactions. Among these, the most prominent interactions are made through αN1 Tyr959 and αJ Met1363, whose side chains are buried within the hydrophobic core at the ‘XX’ interface (Fig. 3c). This intimate packing results in a short-distance H-bond between the hydroxyl groups of Tyr959 from both monomers. The side chain of Met1363 projects from the core to make extensive hydrophobic interactions with αN1 residues Ser962, Leu963, Leu966 and αJ residues Met1364 and Ala1367 (Fig. 3c). Additional hydrophobic interactions at the αN1–αJ interface include those made by αN1 leucines 955 and 966 (Fig. 3d). Interestingly, the side chain of Leu966, which occupies the ‘knob’ position, is protruding into a hydrophobic ‘hole’ formed by αJ residues Met1363, Phe1366, Ala1367 and Val1371. Likewise, hydrophobic residues αN1 Tyr959 and αJ Met1357 and Ala1360 surround the side chain of Leu955, highlighting the importance of leucines as helix-stabilizing residues (Fig. 3d).

**Fig. 4:**
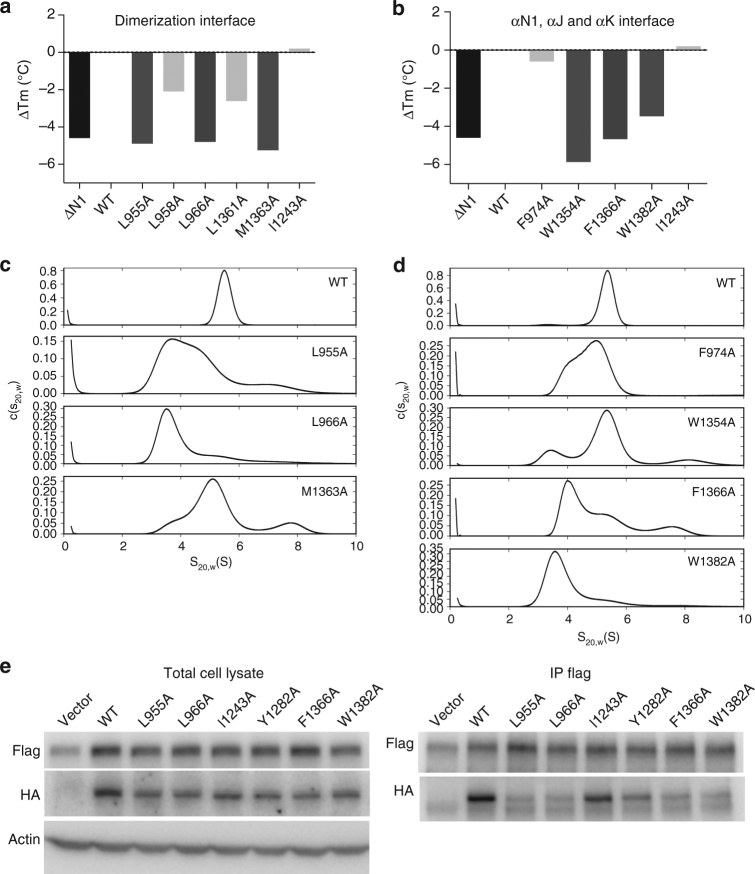
Two clusters of hydrophobic hot spots mediate SgK223 dimerization. **a**, **b** Plots representing the difference in melting temperature between the SgK223-αN1-PsK-Cter (WT), SgK223-PsK-Cter (ΔN1) and various mutants within the regulatory helices. **c**, **d** *c*(*s*_20,w_) distributions for SgK223-αN1-PsK-Cter (WT) and mutants, each analysed at a concentration of 3.0 μM, where the WT protein is primarily dimeric. **e** Co-immunoprecipitation of Flag-tagged version of SgK223 FL WT with HA-tagged SgK223 FL WT or mutant proteins in HEK293 cells. Lysates were subjected to immunoprecipitation with an anti-FLAG antibody followed by Western blotting with anti-FLAG or anti-HA antibodies as indicated. Total cell lysates were also blotted with these antibodies, as well as for actin as a loading control. The data are representative of three independent experiments. See also Supplementary Figs. 3 and 4

To determine the energetic hot spots that contribute to SgK223 dimerization and guide functional studies, we conducted exhaustive alanine scanning mutagenesis of residues within the αN1, αJ and αK helices (Supplementary Fig. 3). The mutants were expressed and purified similarly to WT-SgK223-αN1-PsK-Cter and subjected to TSA to assess the impact of the mutations on the stability of the dimer. We used WT-SgK223-αN1-PsK-Cter that displays a melting temperature (*T*_m_) of 49 °C and SgK223-PsK-Cter without the αN1 helix, which we previously showed to have a significant reduction in thermal stability with a *T*_m_ of 44 °C^12^, to benchmark the impact of individual mutations. Our mutational analysis clearly identified two clusters of hot spot residues that are critical for dimer stability. The first cluster, located at the core of the αN1/αJ dimer interface, consists of residues Leu955, Leu966, Met1363 and Tyr959. Alanine mutation of Leu955 and Leu966 that are central to the core of the αΝ1 helix and Met1363 from the αJ helix whose side chain protrudes within the hydrophobic core, all lead to a markedly lower *T*_m_ (44 °C) compared to WT, indicative of a loss of stability (Fig. 4a). In contrast, alanine mutations of αN1 Leu958 and αJ Leu1361, two residues located further away from the buried interface, do not impact significantly on the thermal stability of the protein (Fig. 4a). Thermal stability analysis of Y959A resulted in a high fluorescent signal at room temperature, suggesting that this mutation leads to drastic increase in exposed hydrophobic patches, a result consistent with the location of Tyr959 at the centre of the hydrophobic core.

The second cluster identified lies between the helices that mediate dimerization and the N-lobe of the PsK domain. Phe1366, in addition to stabilizing Leu966 into a hydrophobic pocket at the dimer interface, forms an important contact at the junction of the αN1, αJ and αK helices within a SgK223 monomer. As expected, F1366A displays a reduced thermal stability (Fig. 4b). Consistent with this, αK Asp1381, which directly interacts with Phe1366 and also makes charged interaction with αJ lysines 1365 and 1369, shows reduced thermal stability when mutated to alanine (Supplementary Fig. 3). Trp1382, which anchors the N-lobe of the PsK domain through its interaction with β3–β4 loop Phe1045 and αN1 Phe974, also shows reduced thermal stability when substituted to alanine (Fig. 4b). Surprisingly, F974A substitution had little effect on stability but it is possible that its substitution is compensated by other interactions at this junction. Substitution of αJ Trp1354 to alanine had a detrimental effect on the stability of the protein highlighting its role in connecting the αJ to the PsK C-lobe (Fig. 4b). Similar to Y959A, R1359A substitution resulted in a high fluorescent signal at room temperature, further highlighting the importance of its side chain protruding internally at the junction of αN1, αJ and αK helices and stabilizing critical hydrophobic interactions within a monomer.

To further examine and validate the importance of these two clusters of hot spot residues on dimer formation, we conducted sedimentation velocity experiments at 3.0 μM, a concentration at which WT SgK223 forms primarily a stable dimer of ~5.6 s (Fig. 4c and Supplementary Fig. 3). At this concentration, the two leucine mutants L955A and L966A show significant shifts of the *c*(*s*) distribution toward lower sedimentation coefficients with respect to the WT protein, with L966A, displaying a major peak at ~3.5 s, indicating a primarily monomeric state. These data confirm the critical role that these leucines play in maintaining the integrity of the hydrophobic core of the dimer interface (Fig. 4c). Of the αN1, αJ, αK interface mutants, αK mutant W1382A is primarily monomeric in solution, exhibiting the largest perturbation of dimerization, while αN1 mutant F974A, despite showing little effect on thermal stability, has a significant impact on dimerization (Fig. 4d and Supplementary Fig. 3). This is consistent with the stabilization role that Phe974 and Trp1382 play in anchoring the dimerization domain to the PsK N-lobe (Fig. 2a). As expected, F1366A has a significant impact on dimerization; however, alanine mutation of Trp1354, which connects αJ to the PsK C-lobe, displayed a smaller shift towards the monomeric form (Fig. 4d). Of note, a number of these mutants show signals at higher sedimentation coefficients than that observed for WT, suggesting the presence of oligomers larger than dimer perhaps due to native oligomerization or non-specific self-association due to exposure of hydrophobic surfaces.

We next interrogated the contribution of the two hot spots of hydrophobic residues on full-length (FL) SgK223 homotypic association in cells. In HEK293T cells, we co-expressed a Flag-tagged version of FL SgK223-WT with FL HA-tagged-WT or mutant proteins characterised as monomeric in our in vitro studies, and studied self-association by co-immunoprecipitation. These experiments revealed that while the differently tagged WT proteins co-immunoprecipitated, as previously described^10^, SgK223 self-association was markedly reduced upon mutations to alanine of the residues Leu955 and Leu966 located at the core of the dimer interface and of the residues Phe 1366 and Trp1382 located at the dimerization-PsK domain junction (Fig. 4e).

In summary, the mutagenesis analysis, combined with co-immunoprecipitation studies clearly identified two clusters of hydrophobic residues that are indispensable for dimer stability in vitro and SgK223 homotypic association in cells. Interestingly, these two hydrophobic hot spots are highly conserved in SgK269 (Supplementary Fig. 3), suggesting that SgK269 will adopt a similar mode of dimerization to that observed for SgK223.

### Role of the αG/A-loop in SgK223 oligomerization

**Fig. 5:**
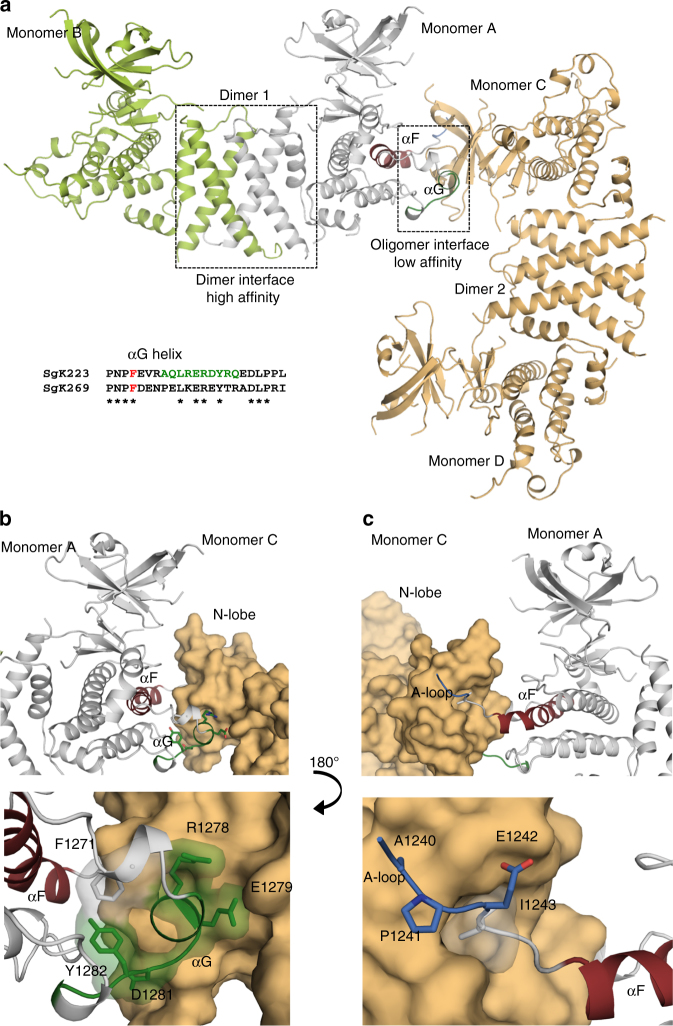
SgK223 oligomerization. **a** Identification of the oligomer interface based on crystal packing between two dimers. **b** Oligomeric interaction made through αG helix (top) and close up of these interactions (bottom). **c** Oligomeric interaction made through the end of the activation loop (top) and close up of these interactions (bottom). Monomer B, lime green; Monomer A, grey; Monomer C and D, pale orange; αG, green; αF, brick red; A-loop, blue. See also Supplementary Fig. 4

Given that the AUC data showed the presence of higher order SgK223 oligomers at concentrations above 18 μM (Supplementary Fig. 1), we investigated which other interfaces could contribute to this oligomerization process by analysing the symmetry related molecules within our crystal packing. Interestingly, analysis through PDBePISA indicated the presence of an interface between the symmetry related dimers, which buries a surface area of 824 Å^2^ (Fig. 5a). Residues from the αF and αG helices and the end of the A-loop contribute to this interface by intimately packing into a groove created by N-lobe residues Leu994, Trp989, Tyr1009, Ile1025, Phe1054, Val1058 and Val1089 of the PsK domain (Supplementary Fig. 4). However, only a few residues from the αG/A-loop site interact directly with the symmetry related dimer (Fig. 5b). The most obvious interaction is through A-loop Ile1243, whose side chain fits snugly into the N-lobe groove to make two main chain H-bonds with Trp989 and Ser1057. In addition, Ile1243, Pro1241 and Glu1242, which mark the end of the ‘APE’ motif of the A-loop, are involved in various interactions with the β4–β5 sheets of the N-lobe of the symmetry related molecule (Fig. 5c; Supplementary Fig. 4).

**Fig. 6:**
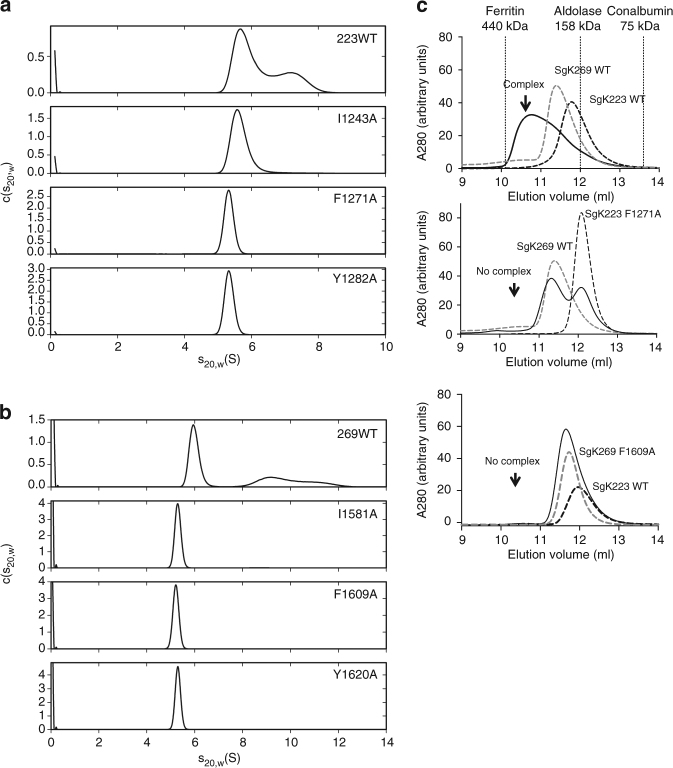
Characterization of SgK223 and SgK269 oligomerization interface. **a** *c*(*s*_20,w_) distributions for SgK223-αN1-PsK-Cter (WT) and mutants at the oligomeric interface, each analysed at a concentration of 20.0 μM, where the WT protein displays significant formation of oligomers larger than dimer. **b** *c*(*s*_20,w_) distributions for SgK269-αN1-PsK-Cter (WT) and mutants at the oligomeric interface, each analysed at a concentration of 20.0 μM, where the WT protein displays significant formation of oligomers larger than dimer. **c** Size-exclusion chromatography analysis (Superdex 200^TM^ Increase 10/300 GL, GE Healthcare) of SgK223 and SgK269 complex. Incubation of SgK223-αN1-PsK-Cter (WT) with SgK269-αN1-PsK-Cter (WT) at 1:1 ratio results in a complex formation (top). Incubation of SgK223-αN1-PsK-Cter F1271A mutant with SgK269-αN1-PsK-Cter (WT) does not form a complex (middle). Incubation of SgK269-αN1-PsK-Cter F1609A mutant with SgK223-αN1-PsK-Cter (WT) does not form a complex (bottom). SgK223-αN1-PsK-Cter (WT) and mutants in black dashed lines; SgK269-αN1-PsK-Cter (WT) and mutants in grey dashed lines; complex of SgK223-αN1-PK-Cter and SgK269-αN1-PsK-Cter in black line. Elution volume of molecular weight standards Ferritin (440 kDa), Aldolase (158 kDa) and Conalbumin (75 kDa) is shown in vertical dotted lines. See also Supplementary Fig. 5

To establish the role of the αG helix/A-loop interface in SgK223 homo-oligomerization, we generated alanine mutations of Phe1271, Arg1278, Glu1279, Asp1281, Tyr1282 and Ile1243. Analysis by SEC indicated that αG-Phe1271 and -Tyr1282 and A-loop Ile1243 mutants showed a sharper elution profile indicative of a reduced amount of higher order oligomers compared to the WT protein (Supplementary Fig. 4). To confirm the absence of higher order oligomers, we conducted sedimentation velocity experiments at 20 μM, a concentration at which WT SgK223 shows self-association and the presence of higher order oligomeric species (Fig. 6a and Supplementary Fig. 5). Sedimentation velocity analysis of F1271A and Y1282A showed loss of higher order oligomers and resulted in *c*(*s*) distributions similar to the WT protein at a concentration of 3.0 μM (c.f. Fig. 4). This indicates that these mutants form stable dimeric structures in solution at raised concentrations. I1243A also showed significant reduction of higher order oligomer formation; however, the *c*(*s*) distribution suggests that a small amount of residual oligomerization remains. To confirm that these oligomerization defective mutants did not perturb formation of the core dimer, we conducted SAXS analysis of I1243A (Supplementary Fig. 5). Data collected on I1243A were monodisperse, as indicated by Guinier analysis, with a maximum particle dimension (*D*_max_) of 130 Å, akin to the WT protein (Fig. 1f), consistent with I1243A existing as a stable dimer in solution.

To further confirm that the oligomerization interface is not a crystal packing artefact, we generated alanine mutants of the corresponding SgK223 αG/A-loop residues in the SgK269-αN1-PsK-Cter construct. As per SgK223 mutants, sedimentation velocity experiments of SgK269 αG (Phe1609, Tyr1620) and A-loop (Ile1581) mutants at 20 μM, showed loss of higher-order oligomers compared to SgK269-WT (Fig. 6b; Supplementary Fig. 5). These data re-iterate the role that the αG/A-loop plays in the formation of SgK223 and SgK269 higher-order oligomeric complexes in vitro.

We next sought to characterize the contribution of the αG/A-loop on FL SgK223 homotypic association in cells. We conducted similar experiments to those described above for the characterization of the dimerization mutants and co-expressed in HEK293T cells a Flag-tagged version of FL SgK223-WT with FL HA-tagged-WT and I1243A or Y1282A mutants. While A-loop I1243A has no detectable impact on self-association, αG Y1282A mutant showed reduced SgK223 self-association (Fig. 4e and Supplementary Fig. 4), indicating that the αG may represent an interface that contributes to SgK223 self-association. However, the impact of the αG mutations on SgK223 homotypic association is less drastic to that observed when mutating the hot spot residues at the dimerization interface and at the junction between the dimerization domain and the PsK domain (Fig. 4e). This result is in agreement with our SEC and AUC data, which indicate that the oligomerization interface is a low-affinity interface (in the μM range) compared to the high-affinity dimerization interface.

### SgK223–SgK269 heterotypic association in vitro

We previously demonstrated, using αN1 and PsK-Cter deletion constructs, a key role for these regions in mediating SgK223-SgK269 heterotypic association in vitro and in cells, a mechanism that is critical for promoting cell migration of MCF-10A mammary epithelial cells and serves to diversify signalling output^10^. Our detailed structure-based mutational analysis, which clearly identified two clusters of hot spot residues that are critical for dimer stability and highlighted a potential role for the αG/A-loop in mediating oligomerization, now allowed us to interrogate the relative contribution of each of these three structural elements to SgK223–SgK269 heterotypic association.

We first sought to investigate the oligomerization interface and if the αG helix F1271A and Y1282A and A-loop I1243A mutants shown to be defective in SgK223 oligomerization in vitro have any impact on heterotypic association of recombinant SgK223- and SgK269-αN1-PsK-Cter proteins using SEC analysis. We previously showed using this technique, that pre-incubation of recombinant SgK223-WT (αN1-PsK-Cter) with SgK269-WT (αN1-PsK-Cter) at a final concentration of 30 μM resulted in a marked shift in their elution profiles compared to when each protein was analysed in isolation^10^. Interestingly, this shift towards the formation of a high molecular weight hetero-complex between SgK223 and SgK269 was not observed with any of the three SgK223 mutants that were shown to be impaired in SgK223 self-oligomerization (Fig. 6c and Supplementary Fig. 5). Similarly, no shift towards the formation of a hetero-complex between SgK223 and SgK269 was observed when recombinant SgK223-WT (αN1-PsK-Cter) was incubated with the SgK269 oligomerization interface mutant αG F1609A (Fig. 6c).

These data further demonstrate the importance of the αG/A-loop in the scaffolding function of SgK223 and SgK269 in vitro. However, it should be noted that the SEC experiments were undertaken by mixing preformed SgK223 and SgK269 homodimers, which, given the high affinity of the dimerization interface compared to that of the oligomerization interface, may bias heterotypic association towards the use of the oligomerization interface.

**Fig. 7:**
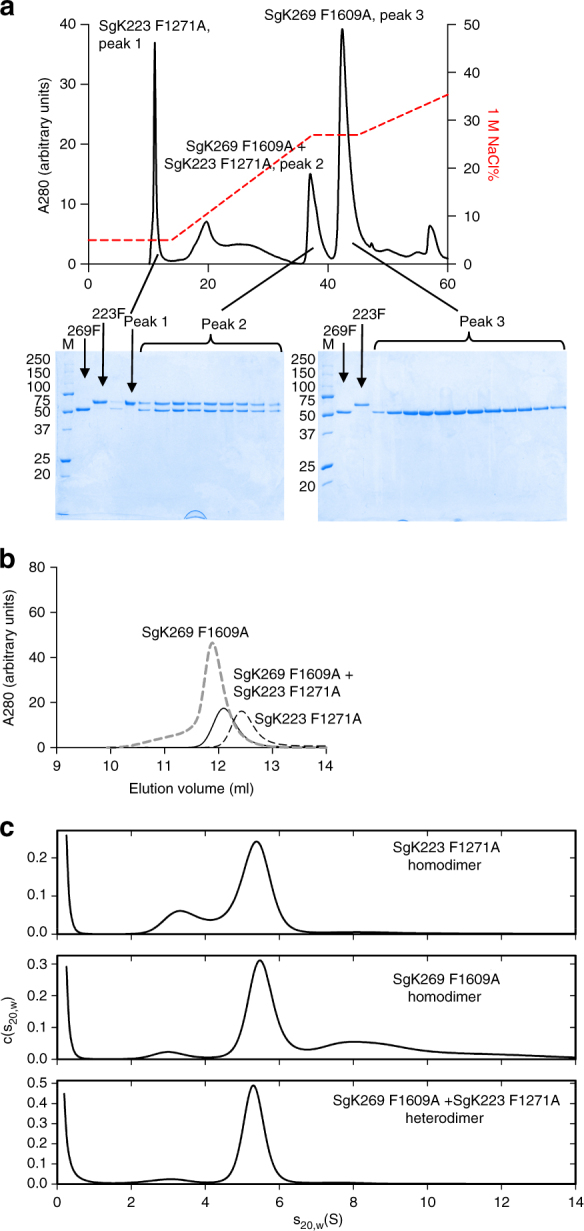
Heterodimerization of SgK223 and SgK269. **a** Mono Q (5/50 GL, GE Healthcare) profile of SgK223-αN1-PsK-Cter F1271A/His tag cleaved SgK269-αN1-PsK-Cter F1609A heterodimer complex. SgK223-αN1-PsK-Cter F1271A and His tag cleaved SgK269-αN1-PsK-Cter F1609A were mixed in equal molar ratio at 20 µM and incubated on ice overnight. The resulting complex was run on Mono Q and eluted by applying 5–60% linear gradient of 1 M NaCl. Protein fractions corresponding to the three main peaks of the homodimer of SgK223-αN1-PsK-Cter F1271A (peak 1), heterodimer of SgK223-αN1-PsK-Cter F1271A and SgK269-αN1-PsK-Cter F1609A (peak 2) and homodimer of SgK269-αN1-PsK-Cter F1609A (peak 3) are shown on an SDS-PAGE along with the molecular weight markers. **b** Size exclusion chromatography analysis (Superdex 200^TM^ Increase 10/300 GL, GE Healthcare) analysis of SgK223-αN1-PsK-Cter F1271A homodimer shown in black dashed line, SgK223-αN1-PsK-Cter F1271A /SgK269-αN1-PsK-Cter F1609A heterodimer shown in black line and SgK269-αN1-PsK-Cter F1609A homodimer shown in grey dashed line. **c** *c*(*s*_20,w_) distributions for SgK223-αN1-PsK-Cter F1271A and SgK269-αN1-PsK-Cter F1609A homodimers in comparison to SgK223-αN1-PsK-Cter F1271A and SgK269-αN1-PsK-Cter F1609A heterodimer, each analysed at a concentration of 1.2 μM. See also Supplementary Fig. 6

To determine whether SgK223 and SgK269 -αN1-PsK-Cter constructs could hetero-dimerize in vitro through the dimerization interface, we exploited the different overall charge properties of SgK223 and SgK269-αN1-PsK-Cter constructs. We reasoned that the SgK223–SgK269 heterodimer should elute at a different salt concentration from an anion exchange column compared to that of the respective homodimers. We used SgK223 and SgK269-αN1-PsK-Cter αG mutants to prevent any oligomerization via the αG, while keeping the dimerization interface intact. Incubation of SgK223-αN1-PsK-Cter F1271A with His-tag cleaved SgK269-αN1-PsK-Cter F1609A was conducted overnight at an equivalent molar ratio to achieve a final concentration of 20 μM prior to loading onto a Mono Q column. These experiments revealed the presence of SgK223–SgK269 heterodimer, eluting at a distinct salt concentration to that of SgK269 homodimer, while His-SgK223-αN1-PsK-Cter homodimer eluted in the flow-through (Fig. 7a). The integrity and molecular weight of the heterodimer purified by Mono Q column, was further confirmed by SEC analysis (Fig. 7b **)**. Sedimentation velocity experiments of the SgK223–SgK269 heterodimer isolated from SEC (Fig. 7b) showed a homogeneous species with a sedimentation coefficient consistent with a dimer (Fig. 7c and Supplementary Fig. 6). The appearance of signal in the *c*(*s*_20,w_) distributions at lower or higher sedimentation coefficients than the dimer peak is attributed to partial degradation of the proteins during the long incubation and processing times required.

### SgK223-SgK269 heterotypic association in cells

**Fig. 8:**
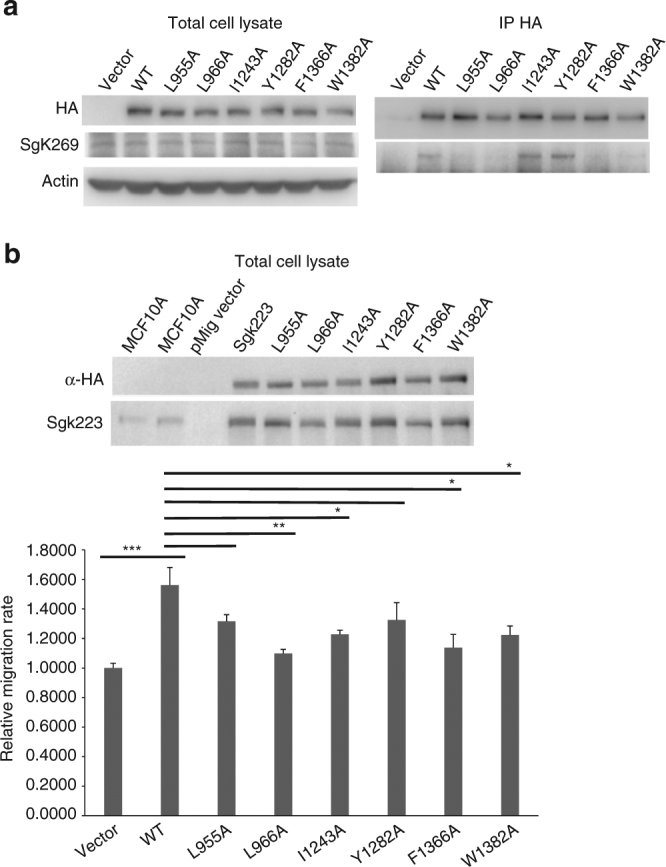
SgK223-SgK269 heterotypic association in cells and SgK223 function. **a** Co-immunoprecipitation of exogenous HA-tagged version of FL WT SgK223 or mutant proteins with endogenous SgK269 in MCF-10A SgK223 KO cells. Lysates were subjected to immunoprecipitation with an anti-HA antibody followed by western blotting with anti-HA or anti-SgK269 antibodies as indicated. Total cell lysates were also blotted with these antibodies, as well as for actin as a loading control. The data are representative of two independent experiments. **b** Effect of interface mutations on SgK223-promoted cell migration. Western blotting of total cell lysates to confirm expression levels of SgK223 FL WT and mutants in MCF-10A parental and SgK223 KO cells. Lysates were blotted with the indicated antibodies (top). Effect of SgK223 FL WT and mutants on cell migration in MCF-10A SgK223 KO cells (bottom). Relative wound closure was measured using TScratch software^59^ and used to calculate the cell migration rate. This is expressed relative to vector control, which was arbitrarily set at 1.0. Data represent mean ± SEM from four independent experiments. ***p < 0.005 (for comparison of WT to vector), *p < 0.05, **p < 0.01 (for comparison of mutants to WT). See also Supplementary Fig. 7

To characterize the contribution of the dimerization and oligomerization interfaces on SgK223–SgK269 heterotypic association in cells, we undertook co-expression studies in SgK223 knock-out (KO) MCF-10A cells in order to remove any potential contribution of endogenous SgK223. The four mutants identified as defective in dimerization (L955A, L966A, F1366A and W1382A) and the two αG mutants identified as defective in oligomerization (I1243A and Y1282A) in vitro were expressed as HA-tagged versions and association with endogenous SgK269 was assayed by co-immunoprecipitation studies (Fig. 8a). These experiments revealed that while HA-tagged SgK223-WT was able to co-immunoprecipitate endogenous FL SgK269-WT, as previously described^10^, SgK223 heterotypic association with SgK269 was abolished with all four SgK223 dimerization mutants. These data suggest that SgK223–SgK269 interaction occurs directly via the dimerization interface, a finding consistent with the demonstrated heterodimerization of SgK223 and SgK269 αG mutants in vitro (Fig. 7) and the high level of conservation of the dimerization domain between SgK223 and SgK269 (Supplementary Fig. 3). In contrast, no detectable impact on SgK223–SgK269 heterotypic association was observed with the αG/A-loop SgK223 mutants (Fig. 8a). However, based on our structural and biochemical data, we cannot exclude a role for the αG/N-lobe in SgK223–SgK269 interaction. The dimerization defective mutations, located at the core of the αN1/αJ dimer interface and at the junction between the dimerization domain and the N-lobe of the PsK domain, are likely to affect the relative orientation of the N-lobe of the PsK domain, and hence the positioning of the αG/N-lobe interface, which may impair association with full-length SgK269 in vivo. In contrast, the mutations introduced on the αG/A-loop would have limited impact on the overall conformation of the PsK domain. Further mutational analysis directed at drastically modifying the αG/A-loop conformation and targeting the complementary PsK N-lobe interface will be necessary to fully interrogate the contribution of the low-affinity αG/A-loop/N-lobe interface, independently from the high affinity dimerization interface, on SgK223–SgK269 heterotypic association in cells.

In order to validate the impact of the dimerization and oligomerization mutations on SgK223 biological function, we utilized cell migration assays, as overexpression of SgK223 enhances cell migration^19^ and SgK223 is required for SgK269 to promote cell motility in MCF-10A cells^10^. Introduction of SgK223-WT or specific mutants into SgK223 KO MCF-10A cells resulted in modest overexpression of the proteins at equivalent levels (Fig. 8b and Supplementary Fig. 7). Expression of SgK223-WT resulted in an ~1.5-fold enhancement of cell migration rate relative to the vector control. However, the ability of SgK223 to enhance migration was significantly reduced for L966A (dimerization interface), F1366A and W1382A (junction between the dimerization domains and the PsK domain) and I1243A (A-loop). A non-significant trend for decreased migration was observed for dimerization mutant L955A and αG helix mutant Y1282A (Fig. 8b). Overall, these results provide strong evidence that the integrity of both the dimerization and oligomerization interfaces is required for optimal promotion of cell migration by SgK223.

## Discussion

In this study, we used X-ray crystallography to provide high-resolution insights into the molecular mechanism that drives SgK223 self-association. Our structure is a remarkable example of how pseudokinases have compromised their nucleotide binding function to evolve as dynamic scaffolds. Despite an overall common kinase fold, the pseudokinase domain of SgK223 displays striking structural differences in its N-lobe when compared to conventional active kinases. SgK223 PsK domain is characterized by an inaccessible ATP-binding site, a structural feature conserved with the previously characterized pseudokinases VRK3, TRB1, ROP2 and ROR2^9, 13, 26, 27^. The occluded ATP-binding site in SgK223 PsK domain is a result of a conformational repositioning of the N-lobe in relation to the C-lobe, driven by a non-canonical interaction between the conserved Lys from the β3-strand and Gln1048 from the loop preceding the β4 strand, an interaction that is further stabilized by the unique Phe1045 (N-lobe), Trp1382 (αK) and Phe974 (αN1) triad (Fig. 2a). Without geometrical constraints required for catalytic activity, the SgK223 pseudoactive site seems to have evolved new interactions to accommodate the positioning of the adjacent structural elements.

Pseudokinase domains, like kinase domains, are often surrounded by accessory regions that provide additional, often regulatory functionalities. However, many aspects of the non-catalytic signalling functions of pseudokinases remain poorly understood. Deciphering the structural diversity of these non-catalytic elements is critical to understand how these catalytically inert modules disseminate or coordinate signal outputs. Recent work on the pseudokinase Mixed Lineage Kinase domain-like (MLKL), a key effector of necroptosis involved in innate immunity and inflammation, elegantly demonstrates how a conformational change of the pseudokinase domain unleashes the N-terminal four-helix bundle domain, subsequently leading to MLKL oligomerization, membrane translocation and permeabilization^7, 28^. Structural studies of the PAN3 adaptor pseudokinase clearly highlights how a coiled-coil region and a helix directly surrounding the PsK domain, drive an asymmetric domain swapped dimer that creates a docking site for the deadenylase PAN2 to form a catalytically competent PAN2–PAN3 deadenylase complex that regulates mRNA degradation^29, 30^. The crystal structure of SgK223 presented here is another remarkable example of the structural and functional diversity of the pseudokinase family. Our structure reveals how the αN1 helix N-terminal to the pseudokinase domain packs against helices in the C-terminal domain in *cis* to form a unique ‘X’-shaped helix bundle, which also nucleates the assembly of a high affinity ‘XX’ dimer. By performing extensive mutagenesis in conjunction with TSA and AUC, we identified two hydrophobic clusters of hot spot residues that contribute to the formation of a high affinity dimer. The strict conservation of these residues in SgK269 (Supplementary Fig. 3) indicates that this dimer packing arrangement is likely to be conserved in SgK269. This unique signal-independent homotypic dimerization, not previously reported in any kinase or pseudokinase structures, is likely to play an important role in promoting the structural rigidity necessary to drive SgK223’s scaffolding function, where the two central PEST regions bring the effectors into close proximity to facilitate their transactivation, in a manner analogous to the cytoplasmic tails of dimerized receptor tyrosine kinases (Supplementary Fig. 8). In support of this model, the SgK223–Csk interaction stimulates Csk kinase activity, most likely through a trans-activation event^18^.

Considering that SgK223 and SgK269 have a distinct repertoire of known effectors, homotypic or heterotypic association is likely to provide a means to regulate signalling complex assembly and signal output^10^. The structural studies presented herein have enabled the identification of the key molecular interfaces that underpin SgK223 self-association and may be critical for SgK223–SgK269 heterotypic association. It is tempting to speculate that the specific dimer arrangement would allow the αG helix, which largely bulges out to solvent, to act as a docking site for the assembly of oligomeric complexes. Several studies support a role of the αG helix/C-terminal A-loop in mediating selective interactions between kinase/pseudokinase domains and substrates/regulators. A classic example of this mode of interaction is the kinase-regulator structure PKA-RIα^31^. This non-catalytic protein interaction function driven by the αG helix is also conserved within the pseudokinase family. The pseudokinase integrin linked kinase (ILK) interacts with its activator α-parvin CH2 for focal adhesion targeting through a protruding surface on the C-lobe that comprises the αEF and αG helices and the C terminus of the activation loop (Supplementary Fig. 8). Mutations of critical residues driving this interaction completely abolished the binding of parvin to ILK in vivo and dramatically impaired ILK localization to focal adhesion sites^11^.

A key issue arising from our studies is the relative contribution of the dimerization and oligomerization interfaces to SgK223/SgK269 homo- and heterotypic association. In the case of SgK223, our data support a contribution from both interfaces for homotypic association in vitro and in vivo. Mutations in either interface negatively impacted the ability of SgK223 to promote cell migration, indicating that dimerization and formation of higher order oligomers is required for biological activity. However, while we could demonstrate a role for both interfaces in SgK223/SgK269 heterotypic association in vitro, and a role for the dimerization interface in heterotypic association in vivo, our co-IP data do not reveal a role for the oligomerization interface in heterotypic association between SgK223 and SgK269 in cells. This indicates that the contribution of the SgK223 oligomerization interface to biological activity is likely reflecting SgK223 homotypic association.

In conclusion, the pseudokinase SgK223 structure presented in this study reveals a novel dimerization domain fold and highlights the remarkable adaptability of the kinase scaffold to accommodate accessory regulatory domains in order to drive non-catalytic functions. The structural identification of the key molecular interfaces that underpin SgK223/SgK269 homo- and hetero-typic association represents a major advance in our fundamental understanding of how pseudokinases function in cell signalling.

## Methods

### Recombinant protein expression and purification

Synthetic genes of human SgK223 αN1-PsK-Cter (residues 932–1406), SgK269 αN1-PsK-Cter (residues 1267–1746), truncated SgK223 αN1 constructs (residues 975–1406) and mutants (GenScript) were cloned into a modified pCOLD IV vector (Takara) that contains a N-terminal His TEV cleavage tag. Recombinant plasmid were used to transform *E. coli* C41(DE3) (Supplementary Table 1). Protein expression was carried out in *E. coli* C41(DE3) strain and expression cultures were grown according to the auto-induction method^32^. For SgK223 purification, cells were resuspended in lysis buffer containing 20 mM Tris pH 8.5, 500 mM NaCl, 10% v/v glycerol, 5 mM imidazole, 0.1% Thesit, 2 mM EDTA and 10 mM DTT, supplemented with Complete EDTA-free Protease Inhibitor Cocktail (Roche) and lysed by sonication. The supernatant was clarified by centrifugation at 45,000×*g*, 4 °C, 30 min, filtered and loaded onto a 1 ml Ni-NTA resin (Roche). After extensive washes with wash buffer (20 mM Tris pH 8.5, 500 M NaCl, 10% v/v glycerol, 5 mM imidazole, 2 mM EDTA and 10 mM DTT), the protein was eluted in the wash buffer supplemented with 150 mM imidazole. SgK223 containing fractions were subjected to size exclusion chromatography (SEC) (Superdex-200 16/600, GE Healthcare) in SEC buffer (20 mM Tris pH 8.5, 200 mM NaCl, 5% v/v glycerol, 1 mM TCEP). SgK269 and mutants were purified using a similar protocol as SgK223 except that the pH of Tris was changed to pH 8.0 during purification and analysis. Fractions containing SgK223 or SgK269 proteins were pooled, concentrated and flash-frozen for storage at −80 °C. All mutant proteins were subjected to the same purification procedure. The seleno-methionine labelled SgK223 was expressed in *E. coli* C3022I strain (NEB) grown in auto-induction medium in the presence of 125 μg ml^−1^ seleno-methionine^32, 33^. The purification of seleno-methionine-labelled SgK223 was similar to the native protein.

### Structure determination

Crystals of SgK223 and of the seleno-methionine substituted SgK223 were obtained by the vapour diffusion method by mixing 150 μl of protein at 5 mg ml^−1^ with 150 μl of reservoir solution containing 1.6 M NaH_2_PO_4_/KH_2_PO_4_ and 0.1 M Hepes pH 7.5. Crystals were flash-frozen in liquid nitrogen in the reservoir solution supplemented with 10% glycerol. X-ray diffraction data for both native SgK223 and SeMet-SgK223 were collected on the MX2 beamline at the Australian Synchrotron. The native crystal diffracted to 2.95 Å resolution. The data were collected at a wavelength of 0.9537 Å using one crystal and a total rotation of 200° with a 1° oscillation range. MAD scan was performed on the SeMet crystal around the selenium edge. The SeMet data sets were collected at wavelength 0.9537 Å (high energy remote) and 0.9796 Å (inflection point) to resolution between 3.4 and 3.5 Å. At each wavelength, two sets of data were collected at two different positions of the crystal in order to minimize radiation damage and improve anomalous and dispersive signal. All diffraction data were processed using XDS^34^ in primitive tetragonal space group. The SeMet data sets collected at each wavelength were merged and scaled using XSCALE^35^. Table 1 summarizes the data-collection statistics.

Automated experimental phasing were carried out using two-wavelength multiple anomalous diffraction (2W-MAD) phasing protocol of Auto-Rickshaw^36^. The input diffraction data were prepared and converted for use in Auto-Rickshaw using programs of the CCP4 suite^37^. FA values were calculated using the program SHELXC. The maximum resolution for substructure determination and initial phase calculation was set to 4.3 Å. All of the seven heavy atoms requested were found using the program SHELXD^38^ and the absolute configuration (hand) of the substructure was determined using the program ABS^39^. The correct space group was identified to P4_3_2_1_2. The occupancy of all substructure atoms was refined and initial phases were calculated using the program BP3^40^. Density modification and phase extension were performed to 3.4 Å resolution using RESOLVE^41^. A partial model (337 residues out of the total number of 450 residues) was produced using BUCCANEER^42^. Further the native data and the partial model were used in MRSAD protocol of Auto-Rickshaw^43^ for phase enhancement and model completion. The resulting model contained 87% of the total number of residues. The model was further improved using manual model building in COOT^44^, with refinement against the native data set at 2.95 Å using PHENIX^45^. The majority of the residues were visible in the structure with the exception of residues present in surface exposed loops: 932–943, 977–983, 1030–1039, 1065–1081, 1159–1207, 1218–1238 and 1336–1345. The final refined model had 97% residues in the favoured region and 3% residues in the allowed region of the Ramachandran plot. The overall structure was validated using MOLPROBITY^46^. All molecular graphics representations were created using PyMOL (The PyMOL Molecular Graphics System, Version 1.7.4.0, Schrödinger, LLC). PDBePISA was used for buried surface area calculation^47^. Sequence alignment was done using Clustal Omega^48, 49^.

### Thermal shift assay

TSAs were performed as described previously^7, 9, 12, 50^, using a Rotor-Gene Q PCR. The proteins were diluted in 20 mM Tris pH 8.0, 150 mM NaCl, 1 mM DTT to 5.3 μM final concentration and assayed in a total reaction volume of 25 μl. SYPRO Orange (Molecular Probes, CA) was used as a fluorescence probe and detected at 530 nm. We used WT-SgK223-αN1-PsK-Cter that displays a melting temperature (*T*_m_) of 49 °C and SgK223-PsK-Cter without αN1 helix, which we previously showed to have a significant reduction in thermal stability with a *T*_m_ of 44 °C^12^. We also included as control an activation loop mutant (I1243A) located away from the dimerization interface. This mutation had no effect on stability, consistent with its location on the opposite side of the PsK domain.

### Analytical size exclusion chromatography

All SEC analyses were performed on a Superdex^TM^ 200 Increase 10/300 GL (GE Healthcare) equilibrated in 20 mM Tris pH 8.5, 200 mM NaCl, 5% v/v glycerol, 1 mM TCEP. For single elution analysis, WT proteins and mutants were prepared at the final concentration of 2.79 mg ml^−1^ (51.6 μM) in a total volume of 110 μl. For complex interaction studies, SgK223 and SgK269 proteins were mixed in equimolar quantity at 29.06 μM final concentration in a total reaction volume of 110 μl, left 15 min on ice, centrifuged (15,800×*g*, 4 °C, 10 min) before loaded onto the column. Protein fractions were analysed by SDS-PAGE.

For heterodimerization studies, SgK223-αN1-PsK-Cter Phe1271Ala and His tag cleaved SgK269-αN1-PsK-Cter Phe1609Ala were mixed in equimolar ratio at 20 μM in a total reaction volume of 1 ml and incubated overnight on ice. The complex was then diluted five times in 20 mM Tris pH 8.5, 50 mM NaCl 5% glycerol, 1 mM TCEP (buffer A) before loading onto a MonoQ (5/50 GL, GE Healthcare) column. SgK223-αN1-PsK-Cter Phe1271Ala and SgK269-αN1-PsK-Cter Phe1609Ala homodimers were separated from heterodimer of SgK223-αN1-PsK-Cter Phe1271Ala and His tag cleaved SgK269-αN1-PsK-Cter Phe1609Ala by applying a linear salt gradient over 60 column volumes starting at 5% of buffer B (20 mM Tris pH 8.5, 50 mM NaCl 5% glycerol, 1 mM TCEP, 1 M NaCl) until 60% B. The homodimers and heterodimers peaks were analysed by SDS-PAGE and loaded onto a Superdex^TM^ 200 Increase 10/300 GL (GE Healthcare) equilibrated in 20 mM Tris pH 8.5, 200 mM NaCl, 1 mM TCEP for analysis before being subjected to analytical ultracentrifugation.

### Analytical ultracentrifugation

Sedimentation velocity experiments were performed using an XL-I analytical ultracentrifuge (Beckman Coulter) equipped with UV/Vis scanning optics. Buffer reference (20 mM Tris-HCl, 150 mM NaCl, pH 8.0) and sample solutions were loaded into 12 mm double-sector cells with quartz windows and the cells were mounted in an An-60Ti 4-hole rotor. All experiments were conducted at 50,000 rpm (201,600×*g*) and 20 °C, and radial absorbance data were collected at appropriate wavelengths in continuous mode. Data were fitted to a continuous sedimentation coefficient distribution [*c*(*s*)] model and converted to standardized sedimentation coefficient (*s*_20,w_) distributions using SEDFIT^51^. The protein partial specific volumes, buffer density and buffer viscosity were calculated using SEDNTERP^52^. Weight average sedimentation coefficients were determined by integration of *c*(*s*) distributions between 2 and 10 s. Molecular weights were calculated from weight average sedimentation coefficients in SEDFIT using the frictional ratio obtained from the fit to the sedimentation velocity data.

### Small-angle X-ray scattering

SAXS data collection was performed at the Australian Synchrotron SAXS/WAXS beamline using an inline gel filtration chromatography setup, as described previously^7, 50, 53, 54^ with the co-flow modification^55^. Summary statistics for data collection and analysis are reported in Supplementary Table 1. A volume of 90 μl of purified recombinant SgK223 αN1-PsK-Cter WT at 2.2 mg ml^−1^ and SgK223 αN1-PsK-Cter I1243A mutant at 3.5 mg ml^−1^ were injected onto an inline Superdex 200 5/150 column (GE Healthcare) and eluted at a flow rate of 0.2 ml min^−1^ via a 1.5 mm glass capillary positioned in the X-ray beam in 20 mM Tris pH 8,5, 200 mM NaCl, 1 mM TCEP, 5% Glycerol at 12 °C. Scattering data were collected on the monodisperse peak (corresponding to the dimeric form) in 2 s exposures over the course of the elution and 2D intensity plots with consistent scatter intensities from the peak of the sizing exclusion chromatography run (a total of four WT or seven I1243A patterns) were radially averaged, normalized to sample transmission, with background subtraction performed using the Scatterbrain software (Stephen Mudie, Australian Synchrotron). Guinier analysis of data was performed using PRIMUS^56^ and indirect Fourier transform with GNOM^57^ to obtain the distance distribution function, *P*(*r*), and the maximum dimension, *D*_max_, of the scattering particle. CRYSOL^58^ was used to calculate theoretical scattering curves from crystal structure atomic coordinates and compare with experimental scattering curves. Comparison of the experimental scatter pattern obtained for SgK223 αN1-PsK-Cter I1243A mutant with the theoretical scatter calculated using the SgK223 αN1-PsK-Cter WT crystal structure coordinates showed concordance (*χ* = 0.984), consistent with SgK223 αN1-PsK-Cter I1243A existing as a stable dimer in solution and adopting a conformation akin to SgK223αN1-PsK-Cter-WT.

### Cell culture and co-immunoprecipitation experiments

HEK 293 and PlatE cells were maintained in Dulbecco’s modified Eagle’s medium (Gibco) supplemented with 10% FCS (Serana). MCF-10A SgK223 KO cells were as previously described^10^ and were maintained in Dulbecco’s modified Eagle’s medium/nutrient mixture F-12 (Invitrogen) supplemented with 5% (v/v) horse serum (Invitrogen), 20 ng ml^−1^ human recombinant EGF (R&D Systems), 0.5 μg ml^−1^ hydrocortisone (Sigma), 100 ng ml^−1^ cholera toxin (Sigma), 10 μg ml^−1^ bovine insulin (Sigma), 50 units per ml penicillin G (Invitrogen) and 50 μg ml^−1^ streptomycin sulfate (Invitrogen). Retroviral infection of these cells, transfection of HEK293 cells, and co-immunoprecipitation studies, were also undertaken as previously reported^10^. Western blot analysis were performed using the following antibody dilutions: Flag (1:1,000), HA (1:1,000), Actin (1:5,000), SgK223 (1:500), SgK269 (1:500) (Supplementary Table 1). Raw uncropped western blot scans are in Supplementary Figs. 4 and 7.

### Wound healing assay

MCF-10A SgK223 KO cells expressing wild-type SgK223 and mutants were seeded in 24-well plates at 2 × 10^5^ cells per well. After 24 h, wounds were made by scraping with a p20 plastic pipette tip across the cell monolayer, and then the cells were cultured with 1 μg ml^−1^ mitomycin C to prevent cell division. Pictures were taken automatically every 0.5 h from 0 to 18 h and from four or five different places in the same well using a Leica AF6000LX Live cell microscope. The percentage of wound area at the different time points was determined using TScratch software^59^ and relative cell migration rate was calculated from the slope of plotting the percentage of wound area to time as described^60^. Student’s *t*-test was used to calculate *p* values.

### Data availability

The structure of SgK223 αN1-PsK-Cter has been deposited in the Protein Data Bank under ID code PDB:5VE6. All other data supporting the findings of this study are available within the paper, and its Supplementary Information files and are also available from the corresponding authors upon request.

## Electronic supplementary material

Supplementary Info
