# Supplementary material for: Structure of SgK223 pseudokinase reveals novel mechanisms of homotypic and heterotypic association
Source: Nat Commun. 2017 Oct 27;8:1157. doi: 10.1038/s41467-017-01279-9 (PMC5660093; doi:10.1038/s41467-017-01279-9)
Supplement: Supplementary file 1 — Supplementary Info [file 41467_2017_1279_MOESM1_ESM.pdf]

Supplementary Table 1. Reagent and resource table.

REAGENT or RESOURCE	SOURCE	IDENTIFIER
Antibodies		
Anti HA High Affinity from Rat	Roche	Cat#11867423001; Lot 11608200
Anti Flag Probe Rabbit Monoclonal	Santa Cruz	Cat#Sc-807; Lot D308
Anti SgK269 Mouse Monoclonal	Santa Cruz	Cat#Sc-100403; Lot H2814
Anti Beta-actin Mouse Monoclonal	Santa Cruz	Cat#Sc-69879; Lot K1715
Anti Pan 14-3-3	Santa Cruz	Cat#Sc-1657; Lot B1313
EZview™ Red Anti-HA Affinity Gel	Sigma	Cat#E6779; Lot SLBP1147V
ANTI-FLAG® M2 Affinity Gel	Sigma	Cat#A2220; Lot SLBQ0998V
Anti Phospho-Stat3 (Tyr705) Rabbit mAb	Cell signalling	Cat# 9145
Anti Stat3 Mouse mAb	Cell signaling	Cat# 9139
Anti SgK223 Rabbit Ab	From Cambridge Research Biochemicals Ltd. described in[1]	
Bacterial and Virus Strains		
E. coli OverExpress C41(DE3) cells	Sigma	CMC0017
E. coli T7 Express Crystal Competent (High efficiency) for seleno-methionine labeling of proteins	NEB	C30221
Chemicals, Peptides, and Recombinant Proteins		
Selenomethionine	AcrosOrganics	Cat# 3211-76-5
SYPRO Orange protein gel stain	Sigma	Cat# S5692
Crystalllisation reagents<br>1.5 M Potassium phosphate monobasic<br>Quik Optimize™	Hampton Research	Cat# HR2-223; HR2-553
Complete EDTA-free Protease Inhibitor Cocktail	Roche/now Sigma	Cat# 05056489001
TCEP	Thermo Scientific	Cat# #77720
Critical Commercial Assays		
N/A		
Deposited Data		
Atomic coordinates	This paper	PDB: 5VE6
Experimental Models: Cell Lines		
MCF-10A EcoR	From Brugge lab (Harvard) described in [2]	
PlatE	Described in [3]	
MCF-10A EcoR Sgk223 Knockout	Described in [4]	
Oligonucleotides		
Sgk223 forward primer 1 for sequencing CACAGGAACAAGATTGTGTGGTTGTC	This paper	
Sgk223 forward primer 2 for sequencing CATTCTGATCTACGAACTGCTGCAC	This paper	
Sgk269 forward primer 1 for sequencing GCCAGAAGGAGAACCAAGGTGTTATGAGC	This paper	
Sgk269 forward primer 2 for sequencing GACGAGAACCCGGAACTGAAAGAGCGTG	This paper	
pCOLD forward primer for sequencing ACGCCATATCGCCGAAAGG	This paper, Takara Bio Inc.	
pCOLD reverse primer for sequencing TGGCAGGGATCTTAGATTCTG	This paper, Takara Bio Inc.	
Recombinant DNA		
pCOLD IV	Takara/Clontech	Cat. #3360_3364

pCOLD 8xHis Sgk223-αN1-PsK-Cter 932-1406	[4]	N/A
pCOLD 8xHis Sgk223-PsK-Cter 975-1406	[4]	N/A
pCOLD 8xHis Sgk269-αN1-PsK-Cter 1267-1746	[4]	N/A
pCOLD 8xHis Sgk223-αN1-PsK-Cter Y952A 932-1406	This paper	N/A
pCOLD 8xHis Sgk223-αN1-PsK-Cter L955A 932-1406	This paper	N/A
pCOLD 8xHis Sgk223-αN1-PsK-Cter L958A 932-1406	This paper	N/A
pCOLD 8xHis Sgk223-αN1-PsK-Cter Y959A 932-1406	This paper	N/A
pCOLD 8xHis Sgk223-αN1-PsK-Cter R965A 932-1406	This paper	N/A
pCOLD 8xHis Sgk223-αN1-PsK-Cter L966A 932-1406	This paper	N/A
pCOLD 8xHis Sgk223-αN1-PsK-Cter K969A 932-1406	This paper	N/A
pCOLD 8xHis Sgk223-αN1-PsK-Cter L973A 932-1406	This paper	N/A
pCOLD 8xHis Sgk223-αN1-PsK-Cter F974A 932-1406	This paper	N/A
pCOLD 8xHis Sgk223-αN1-PsK-Cter N1353A 932-1406	This paper	N/A
pCOLD 8xHis Sgk223-αN1-PsK-Cter W1354A 932-1406	This paper	N/A
pCOLD 8xHis Sgk223-αN1-PsK-Cter M1357A 932-1406	This paper	N/A
pCOLD 8xHis Sgk223-αN1-PsK-Cter R1359A 932-1406	This paper	N/A
pCOLD 8xHis Sgk223-αN1-PsK-Cter L1361A 932-1406	This paper	N/A
pCOLD 8xHis Sgk223-αN1-PsK-Cter M1363A 932-1406	This paper	N/A
pCOLD 8xHis Sgk223-αN1-PsK-Cter M1364A 932-1406	This paper	N/A
pCOLD 8xHis Sgk223-αN1-PsK-Cter F1366A 932-1406	This paper	N/A
pCOLD 8xHis Sgk223-αN1-PsK-Cter E1368A 932-1406	This paper	N/A
pCOLD 8xHis Sgk223-αN1-PsK-Cter D1381A 932-1406	This paper	N/A
pCOLD 8xHis Sgk223-αN1-PsK-Cter W1382A 932-1406	This paper	N/A
pCOLD 8xHis Sgk223-αN1-PsK-Cter W1330A 932-1406	This paper	N/A
pCOLD 8xHis Sgk223-αN1-PsK-Cter I1243A 932-1406	This paper	N/A
pCOLD 8xHis Sgk223-αN1-PsK-Cter F1271A 932-1406	This paper	N/A
pCOLD 8xHis Sgk223-αN1-PsK-Cter Y1282A 932-1406	This paper	N/A
pCOLD 8xHis Sgk269-αN1-PsK-Cter 1267-1746	This paper	N/A
pCOLD 8xHis Sgk269-αN1-PsK-Cter I1581A 1267-1746	This paper	N/A
pCOLD 8xHis Sgk269-αN1-PsK-Cter F1609A 1267-1746	This paper	N/A
pCOLD 8xHis Sgk269-αN1-PsK-Cter Y1620A 1267-1746	This paper	N/A
pMIG Sapphire SgK223-Flag-WT	[4]	N/A
pMIG Sapphire HA-SgK223-WT	[4]	N/A
pMIG Sapphire HA-SgK223-L955A	This paper	N/A
pMIG Sapphire HA-SgK223-L966A	This paper	N/A
pMIG Sapphire HA-SgK223-I1243A	This paper	N/A
pMIG Sapphire HA-SgK223-Y1282A	This paper	N/A
pMIG Sapphire HA-SgK223-F1366A	This paper	N/A
pMIG Sapphire HA-SgK223-W1382A	This paper	N/A
pMIG Sapphire vector	[4]	N/A
Software and Algorithms		
XDS	[5]	
CCP4 suite	CCP4, 1994	6.5.008
Auto-Rickshaw	[6]	
SHELXD	[7]	
ABS	[8]	
BP3	[9]	
RESOLVE	[10]	N/A
BUCCANEER	[11]	N/A
COOT	[12]	0.7
PYMOL	Schrödinger, LLC	1.7.4.0
PHENIX	[13]	1.9-1692
Prism 7		

References

1. Tactacan, C.M. et al. The pseudokinase SgK223 promotes invasion of pancreatic ductal epithelial cells through JAK1/Stat3 signaling. *Mol Cancer* **14**, 139 (2015).
2. Brummer, T. et al. Increased proliferation and altered growth factor dependence of human mammary epithelial cells overexpressing the Gab2 docking protein. *J Biol Chem* **281**, 626-37 (2006).
3. Croucher, D.R., Rickwood, D., Tactacan, C.M., Musgrove, E.A. & Daly, R.J. Cortactin modulates RhoA activation and expression of Cip/Kip cyclin-dependent kinase inhibitors to promote cell cycle progression in 11q13-amplified head and neck squamous cell carcinoma cells. *Mol Cell Biol* **30**, 5057-70 (2010).
4. Liu, L. et al. Homo- and Heterotypic Association Regulates Signalling by the SgK269/PEAK1 and SgK223 Pseudokinases. *J Biol Chem* (2016).
5. Kabsch, W. Xds. *Acta Crystallogr D Biol Crystallogr* **66**, 125-32 (2010).
6. Panjikar, S., Parthasarathy, V., Lamzin, V.S., Weiss, M.S. & Tucker, P.A. Auto-rickshaw: an automated crystal structure determination platform as an efficient tool for the validation of an X-ray diffraction experiment. *Acta Crystallogr D Biol Crystallogr* **61**, 449-57 (2005).
7. Schneider, T.R. & Sheldrick, G.M. Substructure solution with SHELXD. *Acta Crystallogr D Biol Crystallogr* **58**, 1772-9 (2002).
8. Hao, Q. ABS: a program to determine absolute configuration and evaluate anomalous scatterer substructure. *Journal of Applied Crystallography* **37**, 498-499 (2004).
9. Pannu, N.S., McCoy, A.J. & Read, R.J. Application of the complex multivariate normal distribution to crystallographic methods with insights into multiple isomorphous replacement phasing. *Acta Crystallogr D Biol Crystallogr* **59**, 1801-8 (2003).
10. Terwilliger, T.C. Maximum-likelihood density modification. *Acta Crystallogr D Biol Crystallogr* **56**, 965-72 (2000).
11. Cowtan, K. The Buccaneer software for automated model building. 1. Tracing protein chains. *Acta Crystallogr D Biol Crystallogr* **62**, 1002-11 (2006).
12. Emsley, P. & Cowtan, K. Coot: model-building tools for molecular graphics. *Acta Crystallogr D Biol Crystallogr* **60**, 2126-32 (2004).
13. Afonine, P.V. et al. Towards automated crystallographic structure refinement with phenix.refine. *Acta Crystallogr D Biol Crystallogr* **68**, 352-67 (2012).

Supplementary Figure 1

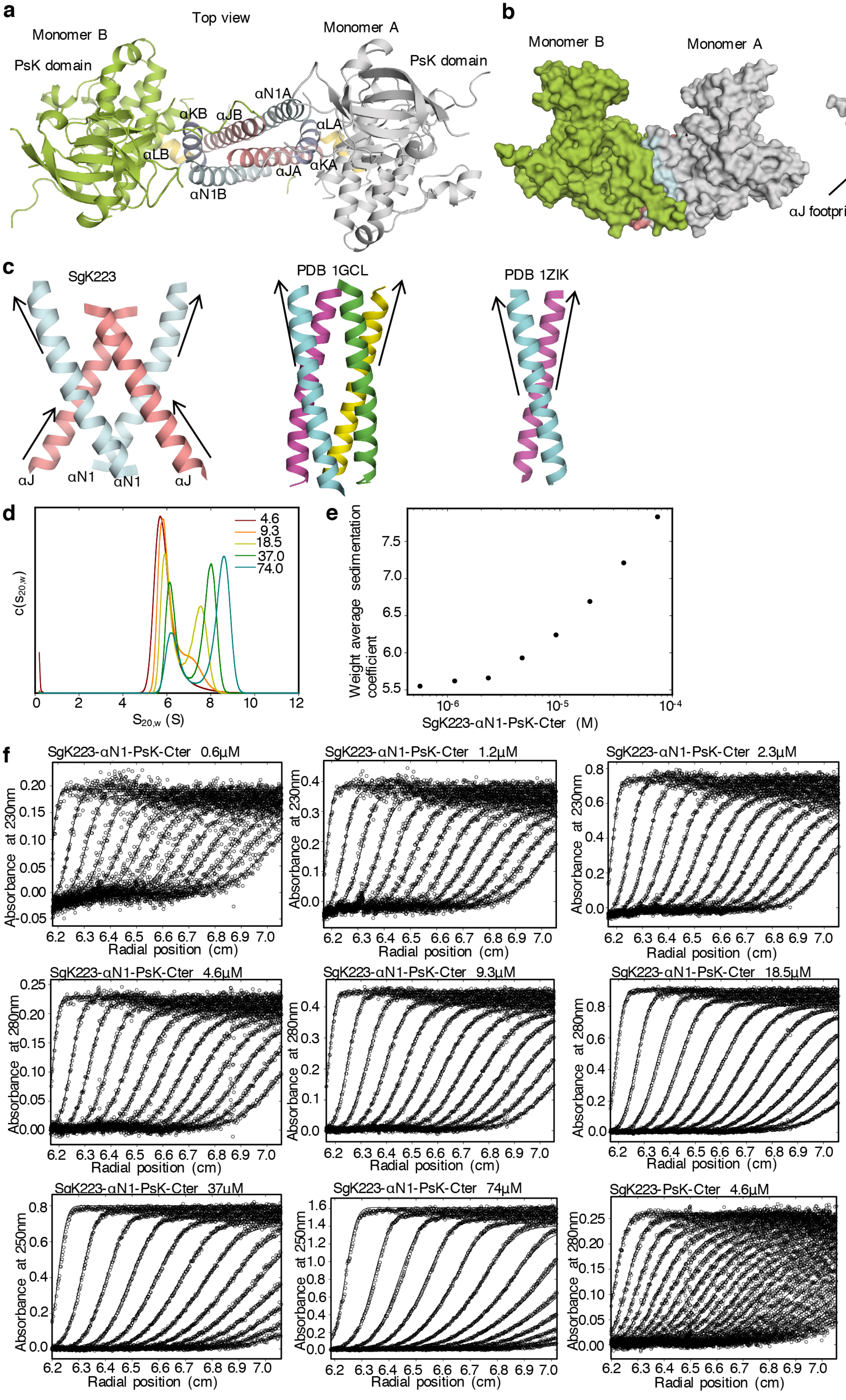

Supplementary Figure 1. Structure of SgK223 and analytical ultracentrifugation data.

(**a**) Top view of the structure of SgK223-αN1-PK-Cter dimer to show arrangement of regulatory helices. (**b**) Surface representation of the dimer to highlight shape complementarity (left) and footprint of αN1 and αJ interaction at the dimer interface. (**c**) Similarity of the unique left-handed arrangement of αN1 and αJ helices at the 'XX' dimer interface to leucine zipper GCN4 (PDB 1ZIK and 1GCL) arrangement. (**d**) Continuous standardized sedimentation coefficient [c($s_{20,w}$)] distributions for SgK223-αN1-PK-Cter analysed at concentrations of 4.6 μM (dark red line), 9.3 μM (orange line), 18.5 μM (light green line), 37.0 μM (dark green line) and 74.0 μM (blue-grey line). Distributions were normalised against integrated signal measured between 2 S and 10 S. (**e**) Standardized weight average sedimentation coefficients for SgK223-αN1-PK-Cter at concentrations between 0.6 μM and 74 μM. Distributions shown in **Fig. 1d and Supplementary Fig. 1d** were integrated between 2 S and 10 S. (**f**) Raw radial absorbance data (circles) are shown overlaid with the best fit to the continuous sedimentation coefficient [c(s)] distribution model (solid lines). SgK223-αN1-PK-Cter analysed at concentrations between 0.6 μM and 74.0 μM, and SgK223-PK-Cter analysed at a concentration of 4.6 μM. Radial absorbance scans collected at 12 minute intervals during sedimentation are shown. See also **Fig. 1**.

a

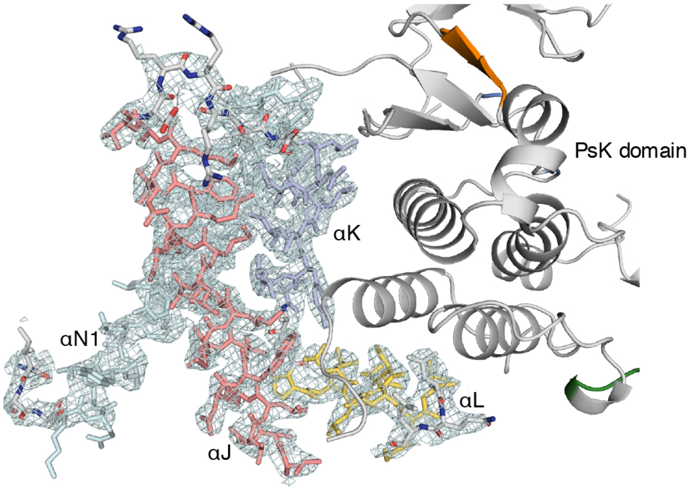

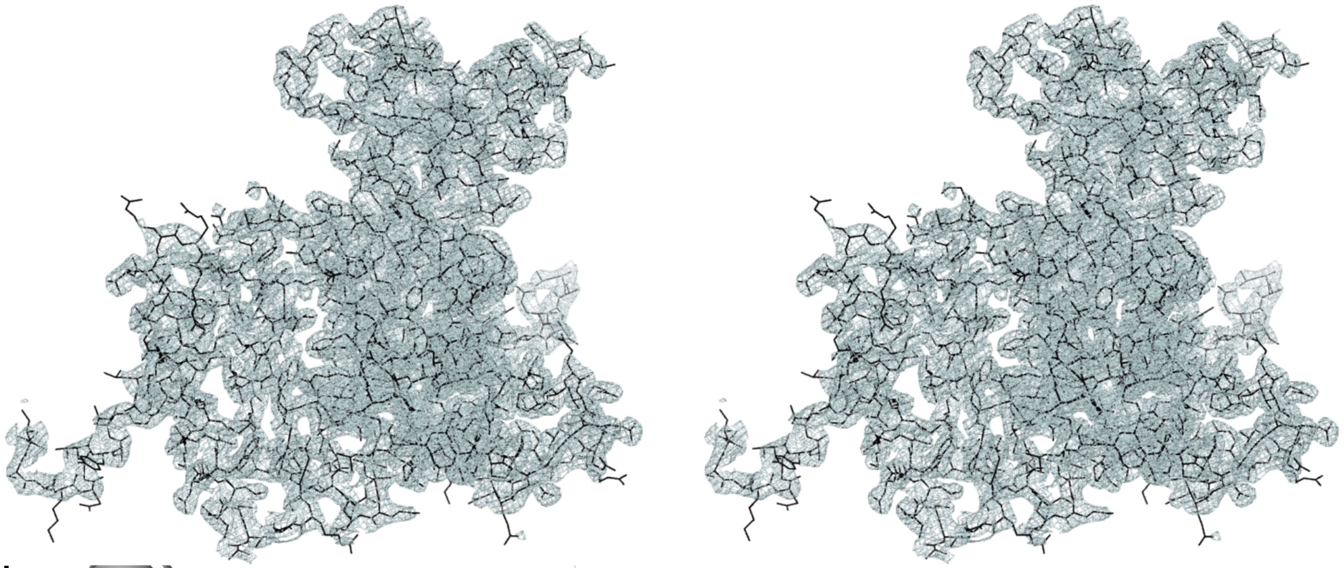

b

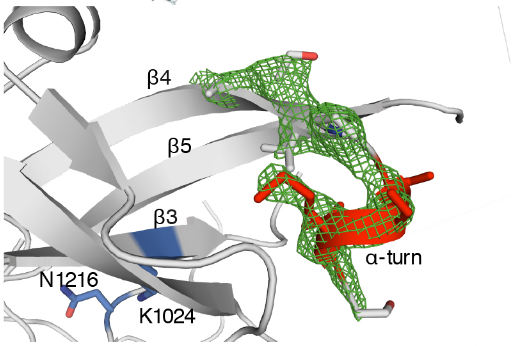

simulated annealing omit map m*Fo*–n*Fc* contoured at 3σ

c

```
               β1            β2            β3          αC
PKA     FDRI--KTLGTGSFGRVMLVKHKE-SGNHYAMKILDKQKVVKLKQIEHTLNEKRILQAVN     93
SgK223  FKLTCNKPCCDSGDAIYYCATCSEDPGSTYAVKICKAPEPKTVSYCSP-------SVPVH   1044
        *                      *  *  ** **
```

Supplementary Figure 2. Electron density maps and αC sequence alignment.

(a) Top, 2*F*o-*F*c map contoured at 1σ of the regulatory helices. Bottom, 2*F*o-*F*c map contoured at 1σ of the overall structure and presented as a wall-eye stereo image. (**b**) The m*F*o–n*F*c map contoured at 3σ was calculated by simulated annealing using SgK223-αN1-PK-Cter, in which α-turn helix was removed. (**c**) Alignment of the αC helix of PKA with SgK223. See also **Fig. 1**.

a

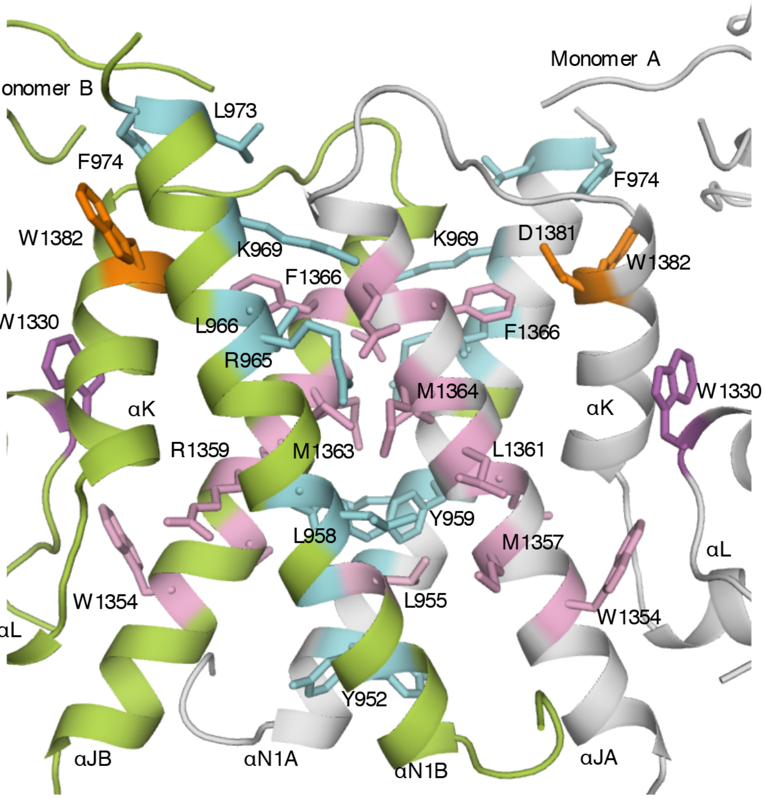

αN1	Y952A, L955A, L958A, Y959A, R965A, L966A, K969A, L973A, F974A
αJ	W1354A, M1357A, R1359A, L1361A, M1363A, M1364A, F1366A
αK	D1381A, W1382A
PsK	W1330A
Control	I1243A

b

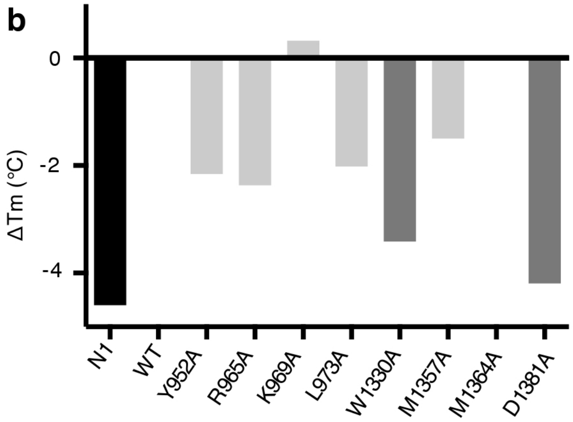

c

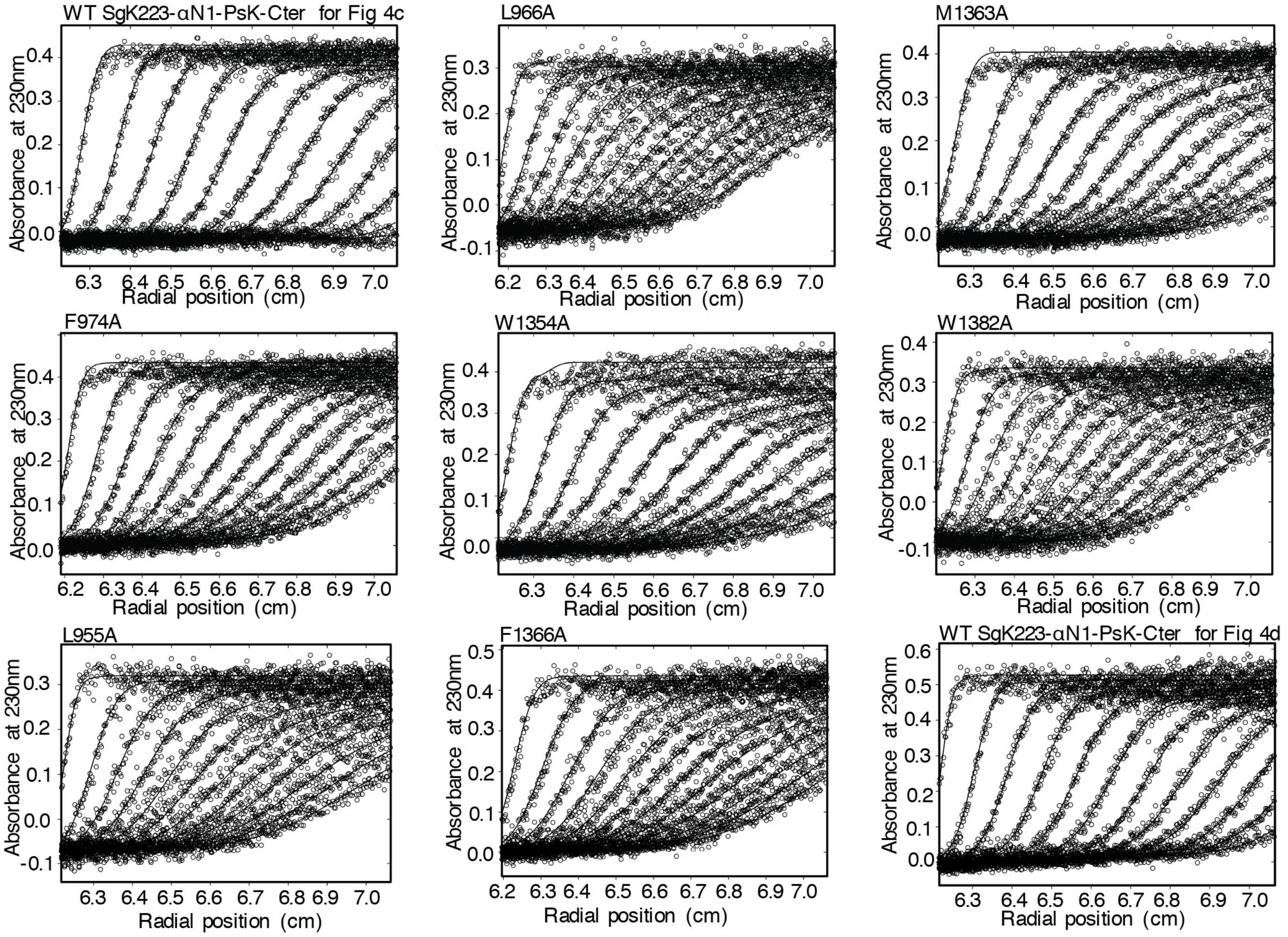

d

αN1

```
SgK223  NISSKEGTYAKLGGLYTQSLARIVAKCEDLFMG   976
SgK269  GLENREEVVGKIRSLHTDALKKIAVKCEDLFMA  1311
             *     *    * *   *    * *******
```

αJ αK αL

```
Sgk223  LCGTLHNWIDMKRAIMMMKFAEKAVDRRRGVELEDWLCCQYLASAEPGALLQSLKLLQLL 1406
SgK269  RNTLLQNWLDIKRTLLMIKFAEKSLDREGGISLEDWLCAQYLAFATTDSLSCIVKILQHR 1746
               * ** * ** * * *****  **  *  ****** *****       *     * **
```

Supplementary Figure 3. SgK223 mutagenesis and sequence alignment with SgK269. (**a**) Location of residues mutated in SgK223. (**b**) Plot representing the difference in melting temperature between the SgK223-αN1-PK-Cter (WT), SgK223-PK-Cter (N1) and various mutants within regulatory helices. (**c**) Raw radial absorbance data (circles) are shown overlaid with the best fit to the continuous sedimentation coefficient [c(s)] distribution model (solid lines). SgK223-αN1-PK-Cter WT and mutants analysed at a concentration of 3.0 μM. Radial absorbance scans collected at 16 minute intervals during sedimentation are shown. (**d**) Conservation of SgK223 and SgK269 αN1 and C-terminal helix region (αN1, αK and αL). See also **Fig. 4.**

Supplementary Figure 4

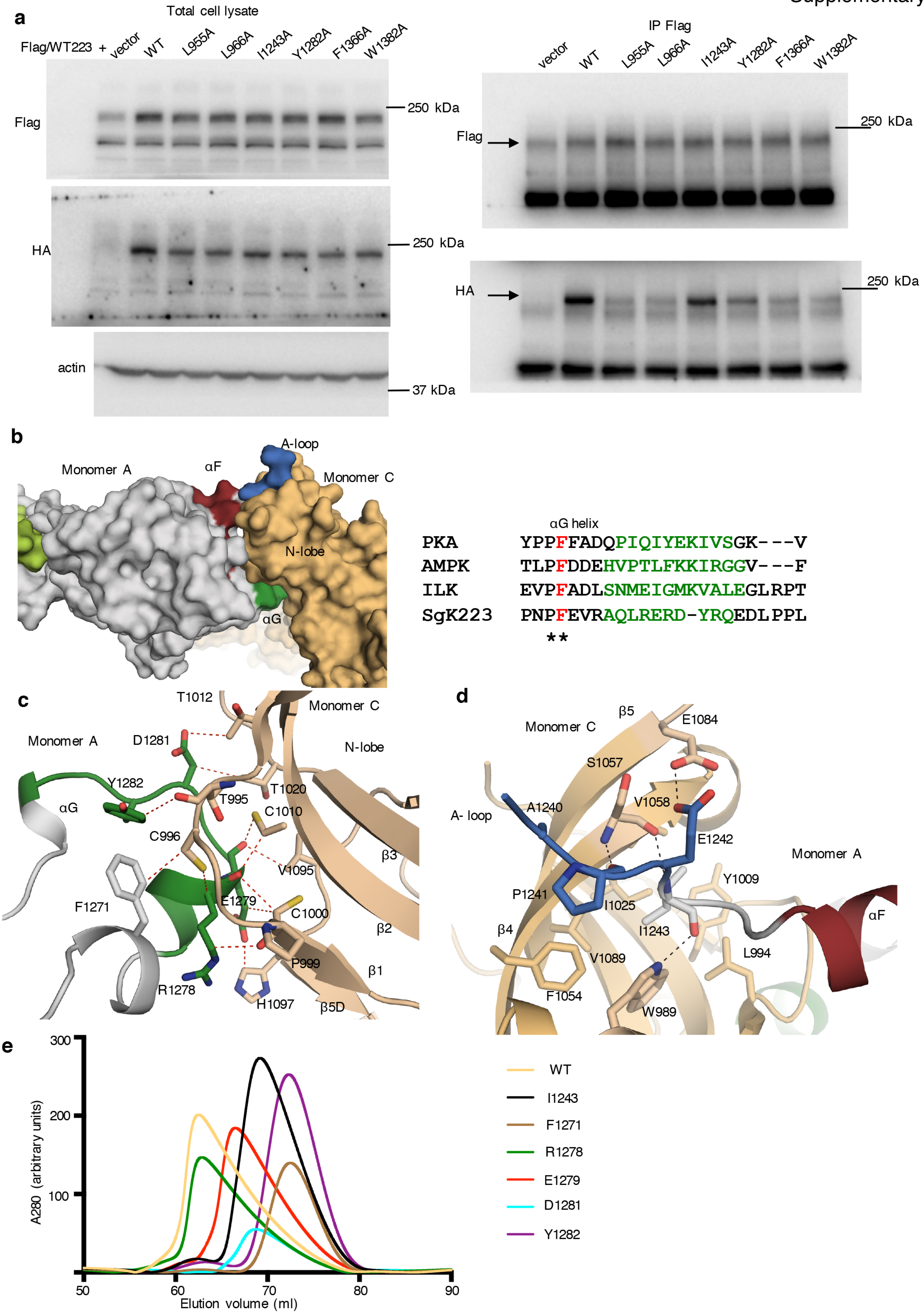

Supplementary Figure 4. Raw western blots, oligomeric interface and size exclusion analysis.

(a) Raw western blots for total cell lysates and co-immunoprecipitation of Flag-tagged version of SgK223 FL WT with HA-tagged SgK223 FL WT or mutant proteins in HEK293 cells (**b**) Surface representation of oligomeric interaction made through αG helix and the end of the activation loop (left and view from the top) and conservation of phenylalanine before the start of αG helix in various kinases and pseudokinases (right). (**c**) Close up of interaction made through αG helix and the N-lobe of the symmetry dimer. (**d**) Close up of interaction made through the end of the activation loop and the N-lobe of the symmetry dimer. Monomer A, grey; Monomer C, pale orange; αG, green; αF, brick red; A-loop, blue. H-bond and van der Waals interactions are shown in black and red dashed lines respectively. (**e**) Size exclusion chromatography analysis (Superdex-200 16/600, GE Healthcare) of SgK223-αN1-PK-Cter WT and mutants to show effect on elution profile by disrupting oligomerization. See also Fig. 4 and **Fig. 5**.

Supplementary Figure 5

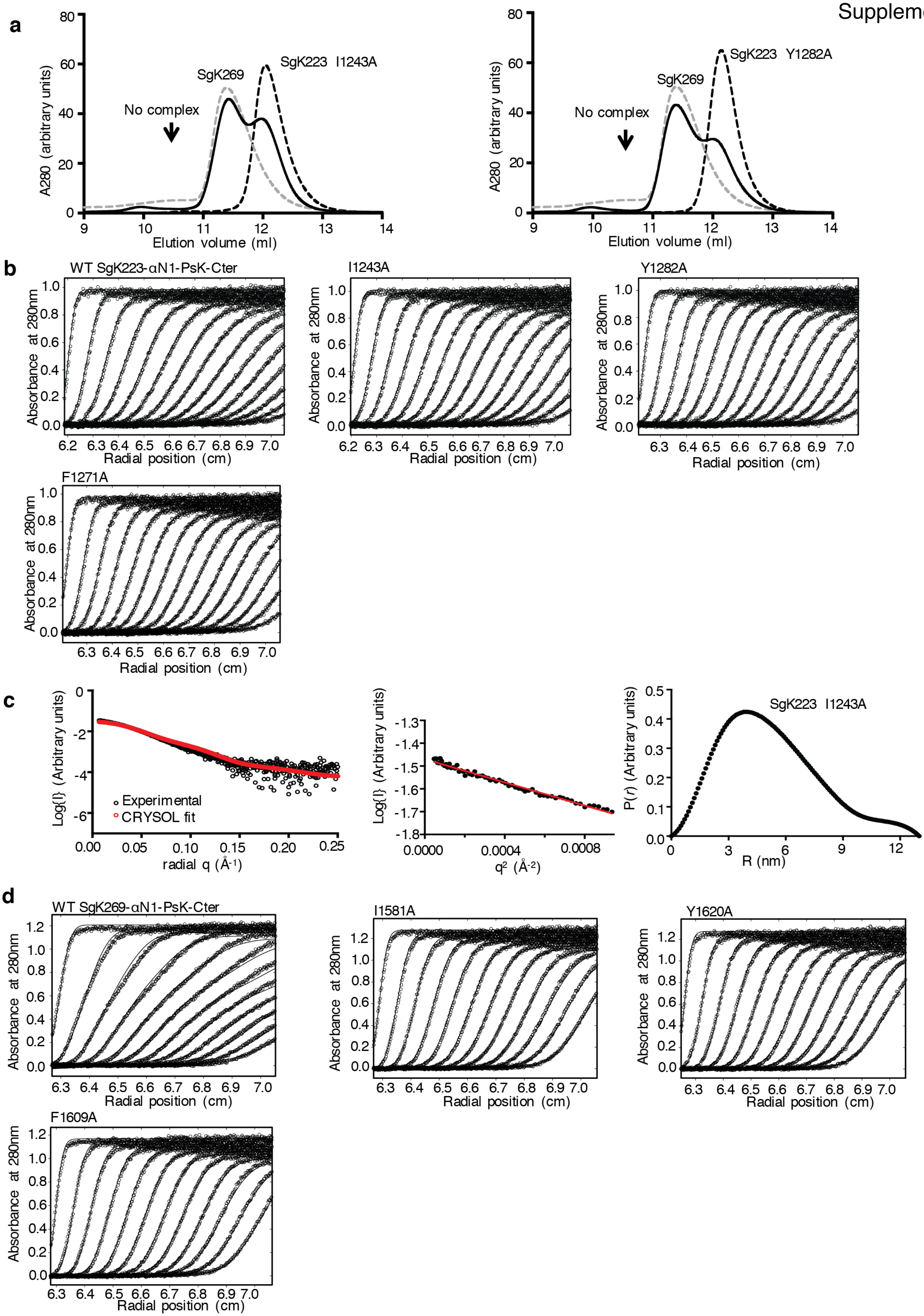

Supplementary Figure 5. SgK223 and SgK269 oligomeric mutants.

(**a**) Size exclusion chromatography analysis of SgK223 mutants and Sgk269 complex. Incubation of SgK223-αN1-PK-Cter I1243A mutant and SgK223-αN1-PK-Cter Y1282A with SgK269-αN1-PK-Cter (WT) does not form a complex. SgK223-αN1-PK-Cter (WT) and mutants in black dashed lines; SgK269-αN1-PK-Cter (WT) in grey dashed lines; complex of SgK223-αN1-PK-Cter and SgK269-αN1-PK-Cter in black line. (**b**) Raw radial absorbance data (circles) are shown overlaid with the best fit to the continuous sedimentation coefficient [c(s)] distribution model (solid lines). SgK223-αN1-PK-Cter WT and mutants analysed at a concentration of 20.0 μM. Radial absorbance scans collected at 12 minute intervals during sedimentation are shown. (**c**) Left: Overlay of experimental scattering data (black circles) of SgK223-αN1-PK-Cter of I1243A mutant and scattering profile calculated using CRYSOL (red). Middle: Guinier plot indicating that aggregates do not measurably contribute to the scattering profile. Right: Interatomic distance distributions. (**d**) Raw radial absorbance data (circles) are shown overlaid with the best fit to the continuous sedimentation coefficient [c(s)] distribution model (solid lines) for SgK269-αN1-PK-Cter WT and mutants analysed at a concentration of 20.0 μM. The lower quality fit to the raw data for SgK269-αN1-PK-Cter WT is attributed to the rapid exchange of higher order oligomers, which are evident as faster moving species in the boundaries. Radial absorbance scans collected at 12 minute intervals during sedimentation are shown. See also **Fig. 6**.

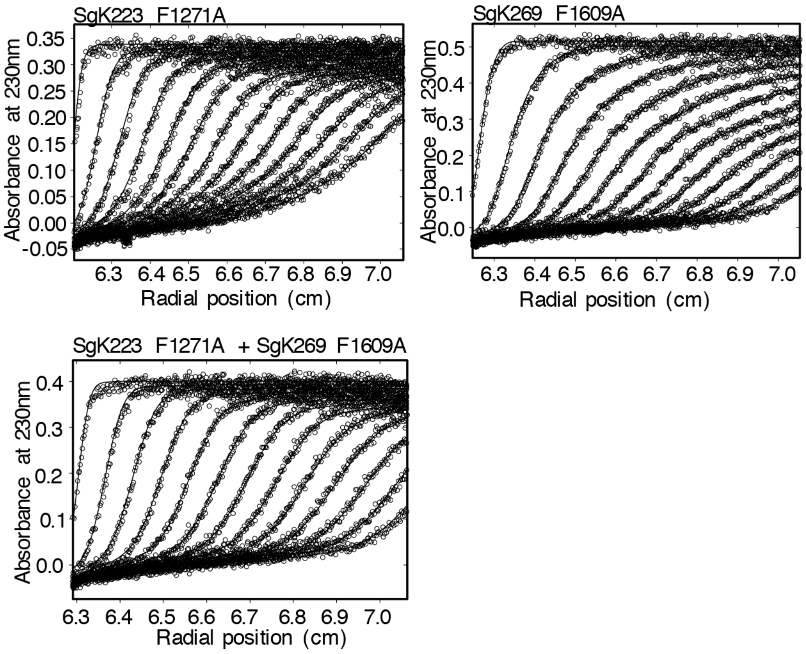

Supplementary Figure 6. Raw radial absorbance data from analytical ultracentrifugation. Raw radial absorbance data (circles) are shown overlaid with the best fit to the continuous sedimentation coefficient [c(s)] distribution model (solid lines) for SgK223-αN1-PsK-Cter F1271A and SgK269-αN1-PsK-Cter F1609A homodimers and heterodimer of SgK223-αN1-PsK-Cter F1271A and SgK269-αN1-PsK-Cter F1609A. All were analysed at a concentration of 1.2 μM. Radial absorbance scans collected at 12 minute intervals during sedimentation are shown. See also **Fig. 7**.

Supplementary Figure 7

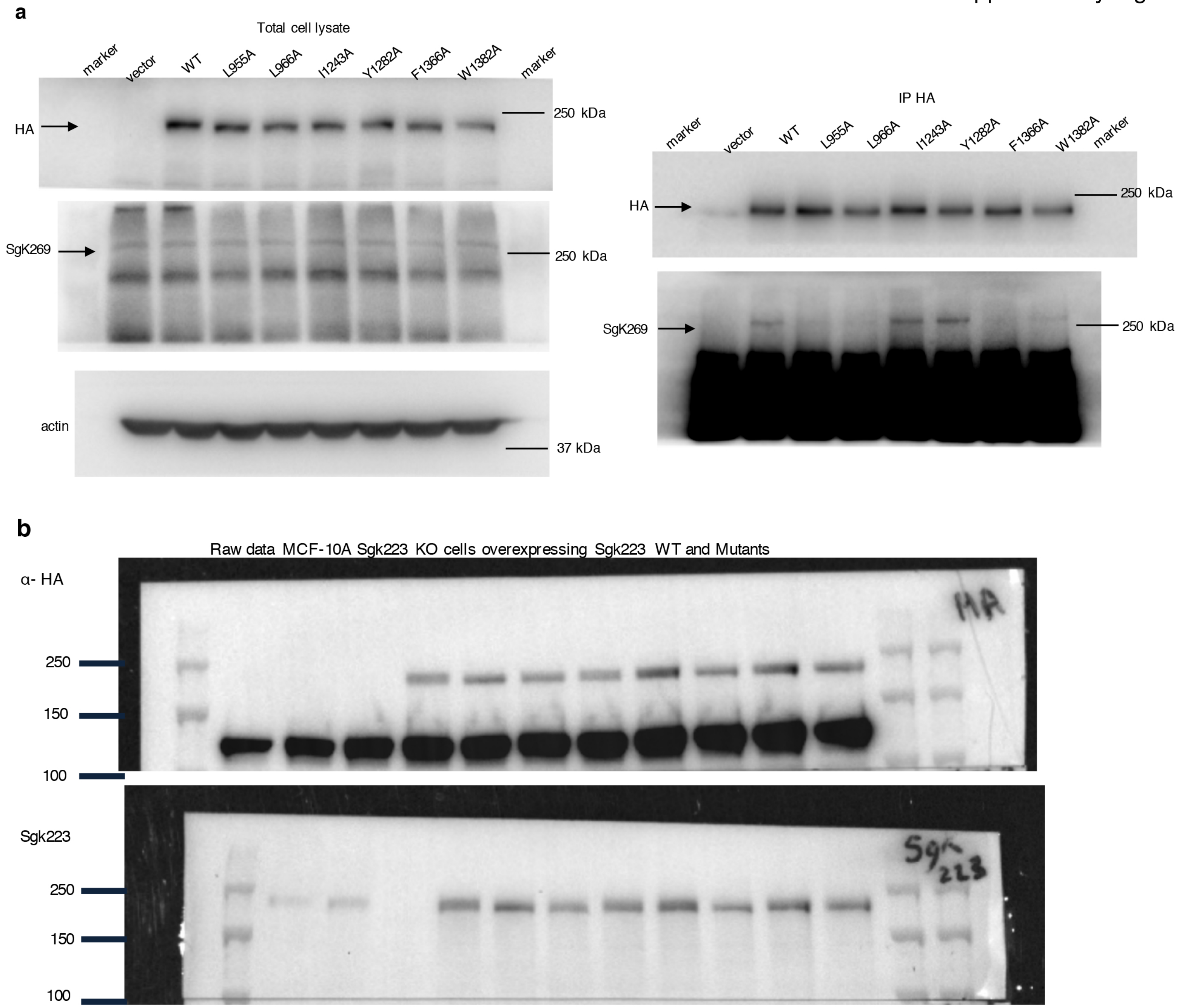

Supplementary Figure 7. **Raw western blots of SgK223 and SgK269 interactions.**

(**a**) Raw western blots for total cell lysates and co-immunoprecipitation of exogenous HA-tagged version of FL WT SgK223 or mutant proteins with endogenous SgK269 in MCF-10A SgK223 KO cells. (**b**) Raw western blots of total cell lysates to confirm expression levels of SgK223 FL WT and mutants in MCF-10A parental and SgK223 KO cells. See also **Fig. 8.**

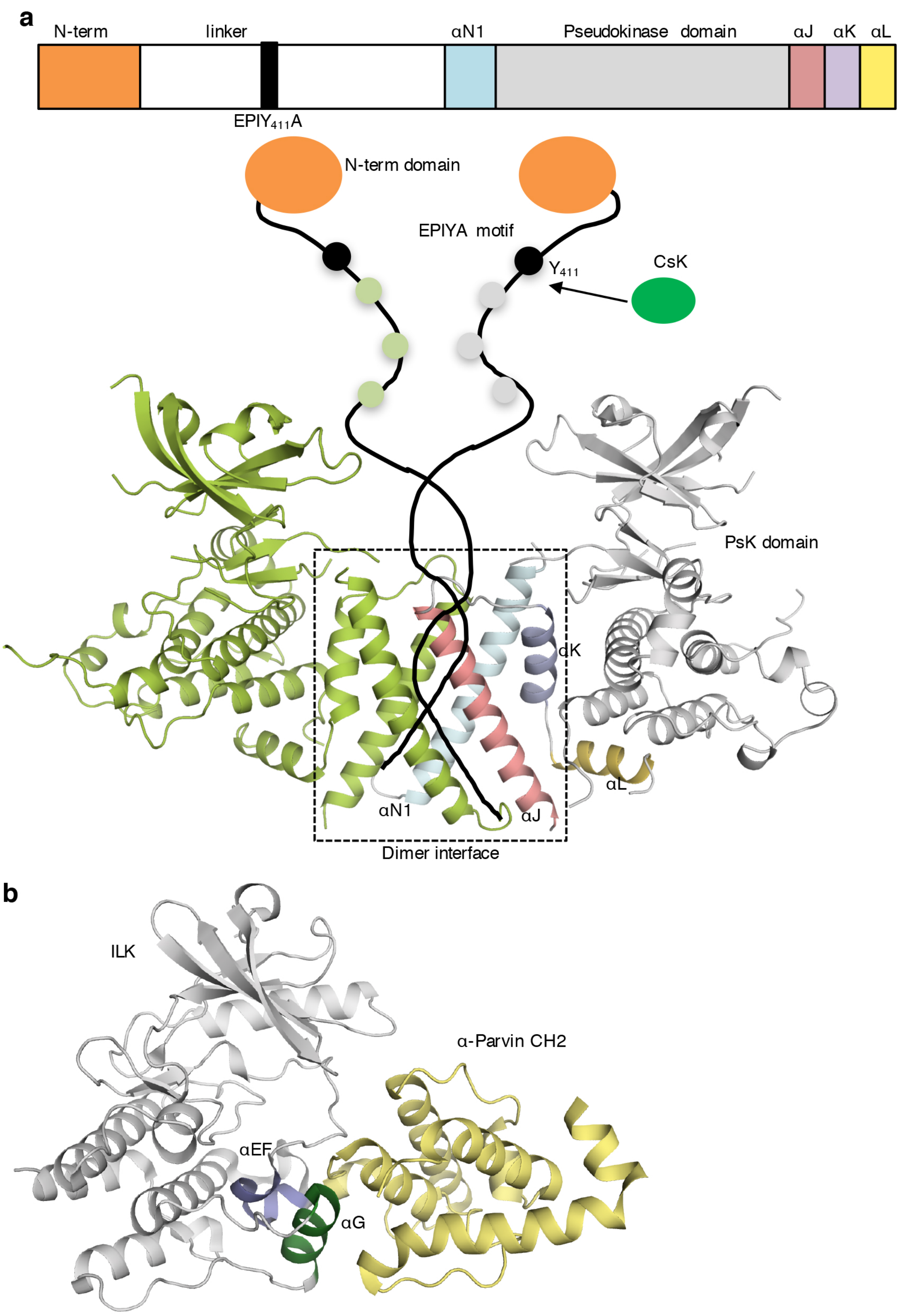

Supplementary Figure 8. SgK dimerization model.

(**a**) Model of SgK223 dimerization. (**b**) Interaction between pseudokinase integrin linked kinase (ILK) and its activator α-parvin CH2 through αG helix.
